# Chemical and photochemical error rates in light-directed synthesis of complex DNA libraries

**DOI:** 10.1093/nar/gkab505

**Published:** 2021-06-22

**Authors:** Jory Lietard, Adrien Leger, Yaniv Erlich, Norah Sadowski, Winston Timp, Mark M Somoza

**Affiliations:** Institute of Inorganic Chemistry, University of Vienna, Althanstraße 14, 1090 Vienna, Austria; European Molecular Biology Laboratory, European Bioinformatics Institute, Wellcome Genome Campus, Hinxton, Cambridge, UK; Erlich Lab LLC, Raanana, Israel; Johns Hopkins University, Department of Molecular Biology and Genetics, Baltimore, MD, USA; Johns Hopkins University, Department of Molecular Biology and Genetics, Baltimore, MD, USA; Johns Hopkins University, Departments of Biomedical Engineering, Molecular Biology and Genetics and Medicine, Division of Infectious Disease, Baltimore, MD, USA; Institute of Inorganic Chemistry, University of Vienna, Althanstraße 14, 1090 Vienna, Austria; Chair of Food Chemistry and Molecular Sensory Science, Technical University of Munich, Lise-Meitner-Straße 34, 85354 Freising, Germany; Leibniz-Institute for Food Systems Biology at the Technical University of Munich, Lise-Meitner-Straße 34, 85354 Freising, Germany

## Abstract

Nucleic acid microarrays are the only tools that can supply very large oligonucleotide libraries, cornerstones of the nascent fields of *de novo* gene assembly and DNA data storage. Although the chemical synthesis of oligonucleotides is highly developed and robust, it is not error free, requiring the design of methods that can correct or compensate for errors, or select for high-fidelity oligomers. However, outside the realm of array manufacturers, little is known about the sources of errors and their extent. In this study, we look at the error rate of DNA libraries synthesized by photolithography and dissect the proportion of deletion, insertion and substitution errors. We find that the deletion rate is governed by the photolysis yield. We identify the most important substitution error and correlate it to phosphoramidite coupling. Besides synthetic failures originating from the coupling cycle, we uncover the role of imperfections and limitations related to optics, highlight the importance of absorbing UV light to avoid internal reflections and chart the dependence of error rate on both position on the array and position within individual oligonucleotides. Being able to precisely quantify all types of errors will allow for optimal choice of fabrication parameters and array design.

## INTRODUCTION

Newly opened avenues in the field of nucleic acid chemistry, from the storage of digital information in DNA (1–3), to DNA origami (4), to supramolecular DNA and gene assembly (5–8), call for high-throughput synthesis of large oligonucleotide libraries. Non-random, parallel synthesis of DNA is the *raison d’être* of microarray fabrication, where synthesis proceeds *in situ* at very large scales, producing anywhere from thousands to a few million unique sequences in a single run. Different approaches allow for oligonucleotide elongation to take place, and while falling into three categories, they all revolve around the use of conventional phosphoramidite chemistry (9–11). In one approach, parallel oligonucleotide synthesis is carried out by controlling the spotting of phosphoramidite solutions onto a surface using an inkjet printer where the CMYK color space is repurposed for the ACGT nucleotide alphabet (12,13), and the removal of the 5′ acid-sensitive protecting group (dimethoxyltrityl, DMTr) blocking the next coupling event occurs simultaneously across the surface. In another approach, this deblocking step is spatially confined using electrochemistry (14) or photogenerated acids (15), and phosphoramidite coupling therefore takes place wherever DMTr groups have been removed. A third approach replaces the DMTr protection strategy with a photosensitive group at the 5′-OH and ultraviolet (UV) light becomes the medium which regulates the next coupling reaction. UV light exposure can be spatially controlled using a Digital Micromirror Device (DMD) on which each micromirror is individually addressable to pattern UV light onto the reactive substrate (16). Such a photolithographic process, when applied to nucleic acid synthesis, was coined Maskless Array Synthesis (MAS) since the DMD replaces the chrome photomasks used in optical lithography.

The throughput of each of these *in situ* DNA array fabrication methods represents a massive improvement over conventional solid-phase synthesis. However, throughput alone is insufficient without control over synthesis accuracy, in particular for applications such as *in vivo* gene administration and digital DNA storage. Libraries obtained by solid-phase synthesis are unlikely to be pure because of incomplete chemical reactions or damage/degradation occurring during or post-synthesis. They are likewise unable to undergo standard purification procedures owing to the minute amounts of surface-synthesized DNA (17–19). Understanding the source and rate of error in the fabrication of complex, high-density nucleic acid libraries therefore becomes key to produce and reach the highest oligonucleotide quality. Inkjet-printed arrays (produced by Agilent and Twist Biosciences) rely on an acidic treatment performed at each cycle to remove the DMTr groups. Assuming 100% effectiveness of coupling, oxidation and deblocking, the hard limit to oligonucleotide length is acid-mediated depurination which can occur at each deblocking event, and is at a statistically higher risk to take place as the oligonucleotide length and number of purines bases increase. The depurination rate can be controlled and lowered by chasing the acidic solution with the I_2_/H_2_O-based oxidizer whose pyridine content quenches the trichloroacetic acid (20). As this limit was pushed further away, 150-nt long oligonucleotide arrays became feasible, with error rates calculated at ∼0.8% per base pair (5) and now reported to be as low as 0.3–0.6% per bp. Employing the photogenerated acid approach for array fabrication yields DNA of slightly lower fidelity, with error frequencies rated at 0.6–1.4% per bp (21,22). High error rates do not necessarily prevent downstream applications, as errors can be accommodated or minimized through purification and selection procedures, most notably by PCR, high-throughput sequencing or with proofreading enzymes (23,24). Modern high throughput digital information storage and transmission are intrinsically error prone, but computer scientists have developed mathematical tools for correcting errors, which can and have been applied to information stored in nucleotide format (25). We recently showed that Reed-Solomon error-correcting algorithms were able to perfectly retrieve the contents of a file stored on DNA oligonucleotides synthesized by MAS, despite a very high synthesis high error rate, at the cost of having to increase information redundancy (26). Indeed, we had employed our express route to photolithographic DNA synthesis (27) which strongly favors speed and economy over fidelity, tolerating incomplete chemical reactions, particularly at the level of photodeprotection. Non-quantitative photodeprotection prevents the next coupling event from being complete, introducing a deletion error in the sequence. Optical effects such as diffraction and scattering play a major role in MAS since light is the controlling element in patterning oligonucleotide synthesis on the surface of the array and we have previously investigated and identified the principle sources resulting in imperfect optical imaging (28). Unintended exposure of the surface originating from the imperfections and fundamental limitations of the optical system will lead to premature removal of the 5′-photosensitive protecting group and hence to an unintended phosphoramidite coupling, *i.e*. an insertion error. Finally, the coupling reaction itself, though extremely efficient chemistry with a 99.9% stepwise yield, remains a source of error which can lead to an omitted coupling and a substitution error if left unaddressed by not including capping chemistry and length filtering.

Altogether, there is a lack of data on the sequence fidelity of libraries synthesized with a light-directed approach as well as a need for an understanding of the combined and complex effects of deletion/insertion/substitutions errors, as already raised in our previous work on MAS accuracy. In this study, we investigated the chemical and photochemical error rate of large oligonucleotide libraries synthesized by MAS as well as the relationship between the different types of error rate and the physical location on the array. To do so, we leveraged the Illumina next-generation sequencing (NGS) platform to estimate the insertion, deletion, substitution and coverage rates and overlaid the information on top of the array layout, and as a function of the position within each oligonucleotide. Although highly reliable, Illumina sequencing is not exempt of errors (29). However, this rate can be considered negligible compared to the expected synthetic error rate. The error rates identified and measured by NGS can be explained in terms of synthesis and photochemical efficiencies, but they also revealed how errors are heterogeneously distributed across the surface of the array, information which will be crucial for optimizing future light-directed fabrication of nucleic acid libraries.

## MATERIALS AND METHODS

### Sequence and microarray design

**Figure 1.**
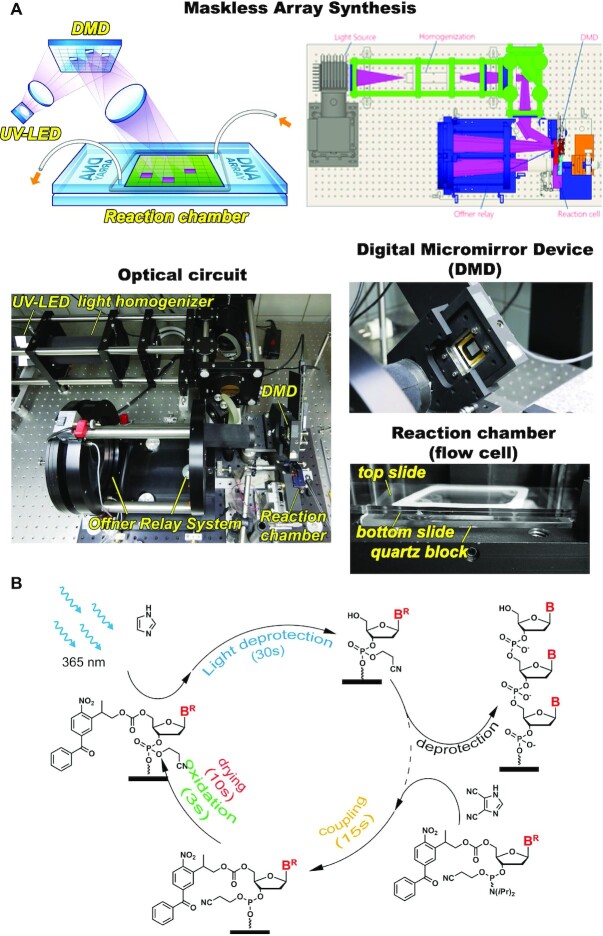
Schematic overview of Maskless Array Synthesis of DNA libraries. (**A**) Photolithography setup showing the principle of patterned UV illumination of the flow cell where oligonucleotide synthesis is taking place, and the optical system of MAS. 365 nm UV light generated by an LED is spatially homogenized and then imaged onto the DMD. Reflections from the ON mirrors are imaged by the Offner relay onto a surface in a reaction chamber connected to a standard solid phase synthesizer. The reaction chamber is composed of two superimposed glass slides separated by a thin gasket, assembled onto a quartz block. The reaction chamber is then attached to an automated DNA synthesizer (not shown). (**B**) The oligonucleotide coupling cycle used for array photolithography from 5′-BzNPPOC DNA phosphoramidites.

Oligonucleotide sequences were designed so as to represent all possible 7-nt long *k*-mers multiple times while avoiding long homopolymers, which resulted in a library of 20 000 unique oligonucleotides (2SZ, capped 2SZ and 4SZ libraries). A more detailed description of the design as well as the full analysis to generate the library are available at: https://github.com/a-slide/DNA_photolitography_seq. A much larger library of 300 000 sequences was also generated (CB_120) with 3′ and 5′ sequencing adapters designed to be synthesized alongside the core oligonucleotide section. Each oligonucleotide sequence included a base-sensitive dT nucleotide at the 3′ end which allows cleavage from the surface (18). A text file containing all sequences was then loaded into a MatLab program, which transforms the list into a series of one-bit bitmap files that represent the pattern of ON and OFF mirrors on the DMD, hence serving as virtual photomasks for the successive photodeprotection events. During synthesis, ON mirrors are tilted to reflect light into an optical relay that images the pattern of light to the array surface (Figure 1A). Each 14 × 14 μm mirror of the XGA mirror array (1024 × 786) is imaged onto the surface with unit magnification resulting in one pixel of synthesis per mirror. Sequences were randomly distributed throughout the surface of the array, with a regular layout of features 4 × 4 pixels in size spaced by 2 pixels or, for the spread-out pattern, features 2 × 2 pixels in size separated by 4 pixels of unused space, resulting in the same number of actual features (20000 for 2SZ and 4SZ). For the CB_120 library, the 300 000 oligonucleotides were randomly distributed into half of the surface pixels following a checkerboard pattern.

### Microarray synthesis

MAS follows protocols that have been established and optimized over the last years for DNA (30–33), as well as extended to other nucleic acids (34–37). Standard glass microscope slides (Schott Nexterion Glass D) were functionalized with *N*-(3-triethoxysilylpropyl)-4-hydroxybutyramide (Gelest SIT8189.5) and for dual-array synthesis, one slide was drilled with a 0.9 mm diamond bit using a CNC router (Stepcraft) prior to silanization. A pair of drilled and undrilled slide was assembled, separated by a 50 μm thick PTFE gasket, into the synthesis cell. The back chamber of the synthesis cell, defined by a 250 μm thick Chemraz 584 perfluoroelastomer gasket separating the slides from a quartz block with fluidics ports, was filled with a 0.5% solution of β-carotene in dichloromethane (CH_2_Cl_2_). All gaskets were custom made with a K40 CO_2_ laser cutter. Finally, the assembled reaction cell was connected to an automated DNA synthesizer (Expedite 8909, Applied Biosystems) delivering reagents (activator: 0.25 M DCI in ACN, exposure solvent: 1% imidazole in DMSO, oxidizer: 20 mM iodine in THF/H_2_O/pyridine 11:2:4), solvent (ACN) and phosphoramidites (Bz-NPPOC dA, dC, dG and dT, Orgentis) to the reaction chamber. Reagents and solvents were from Biosolve. The DNA synthesizer is connected to a computer which controls both the micromirrors of the DMD (Texas Instruments Discovery 1100) and the illumination of the DMD from a UV LED source. An electrical signal from the synthesizer triggers the computer to send the corresponding digital bitmap mask to the DMD and to initiate UV exposure. After a defined period of time corresponding to a total radiant exposure of 3 J/cm^2^, a second electrical signal ends the exposure. DNA phosphoramidites were prepared as 0.03 M solutions in ACN and coupled for 15 s, the NPPOC cleavable dT unit was prepared as a 0.05 M solution in ACN and coupled for 2 × 120 s. DNA phosphoramidites are coupled in the order A→C→G→T then back to A. For the capped library, capping was carried out by an attempted coupling with a DMTr-protected dT phosphoramidite (60 s) after each regular coupling. Since MAS has no detritylation step, the DMTr phosphoramidite effectively caps unreacted 5′-OH groups. The entire synthesis cycle is described in Figure 1B.

### Library cleavage, preparation and sequencing

After synthesis, the microarray slides were deprotected in dry EDA/toluene 1:1 for 2 h at r.t. The arrays were then rinsed with dry ACN (2 × 20 ml) and the cleaved DNA was recovered by applying 100 μl of MilliQ-H_2_O over the synthesis area. The solution was evaporated, rediluted into 10 μl MilliQ-H_2_O then desalted on C18 ZipTips (Millipore), and finally quantified on a Nanodrop Spectrophotometer (Thermo Scientific). Samples were all prepared using the Accel-NGS 1S Plus DNA Library Kit from Swift Biosciences. We used 5 ng of each sample as input for the library preparation protocol following the manufacturer's instructions. The resulting dsDNA libraries were PCR amplified, then analyzed on a TapeStation or Bioanalyzer instrument (Agilent). In order to multiplex the samples for sequencing we used the following barcodes: O1 (index 2) = normal DNA synthesis parameters; O2 (index 4) = cap protecting step between each iteration; O3 (index 5) = increase space between synthesis clusters. The final libraries were sequenced using a MiSeq Instrument and v2 Nano cartridge following the manufacturer's instructions in paired-end mode (2 × 150 bp).

### Sequencing data and error Rate Analysis

Briefly, FASTQ files were obtained and demultiplexed with Illumina Casava bcl2fastq2 Conversion Software v2.20 and adapters were trimmed off using Cutadapt (v1.1.18) to a minimal read length of 20 bases. Reads were subsequently aligned to the sequence panel reference previously generated using Bowtie2 (v2.3.4.3), then sorted and indexed with samtools (v1.9). Finally, we performed the error rate analysis overlaid on the flowcell layout or as a function of the reference base position using a custom python script via a Jupyter Notebook. A more detailed description of the analysis as well as the analysis Notebook and the raw files generated are available at: https://github.com/a-slide/DNA_photolitography_seq. The sequencing data were deposited on ENA under the following project id: PRJEB43002 (https://www.ebi.ac.uk/ena/browser/view/PRJEB43002).

## RESULTS

### Synthetic and photochemical error rates: 20 000-sequence libraries

**Figure 2.**
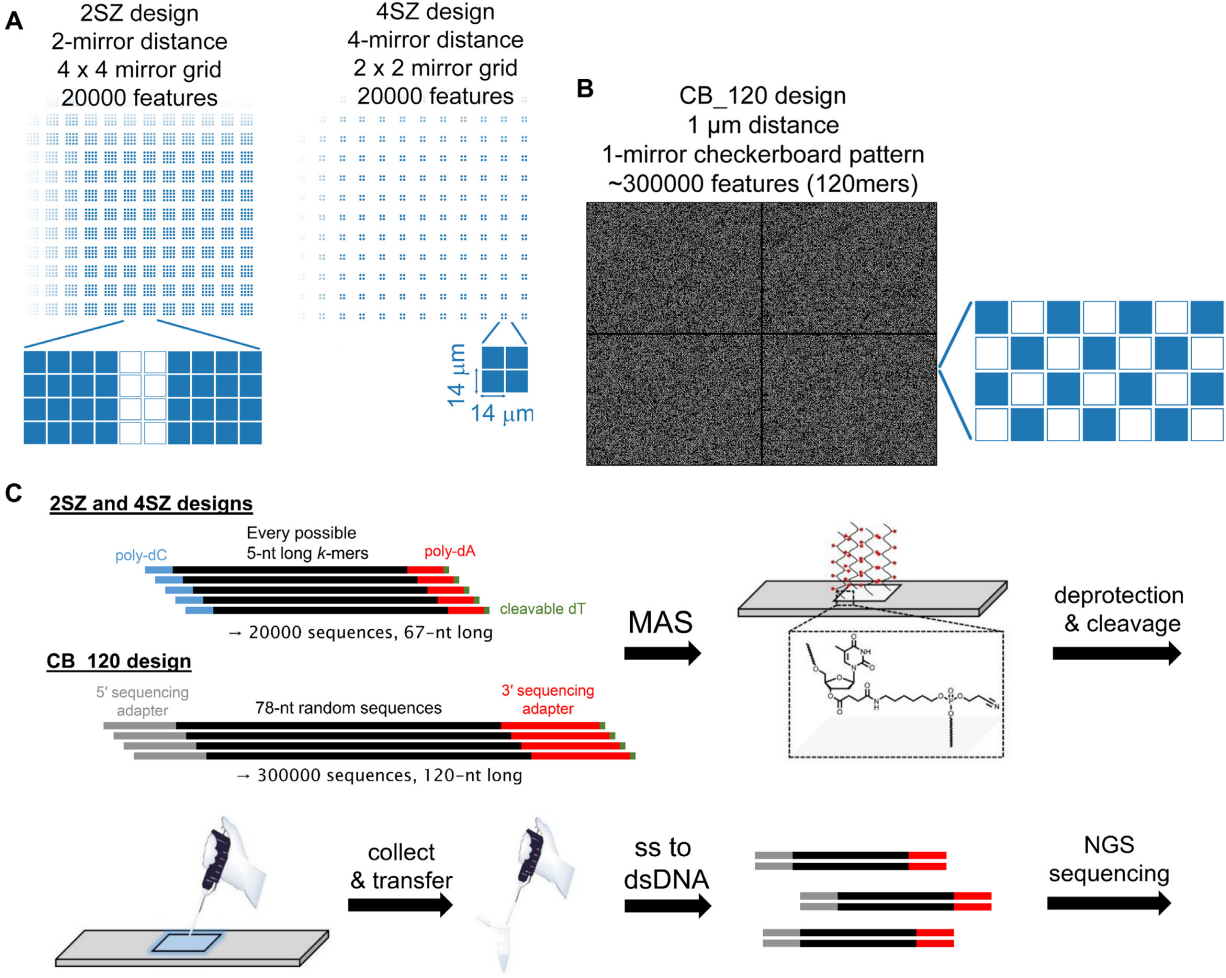
(**A**) Schematic representation of the DNA library designs for MAS: features 4 × 4 mirrors large spaced by 2 mirrors (2SZ) or features 2 × 2 mirrors large spaced by 4 mirrors (4SZ). Two 4 × 4 mirror grids separated by a 2-mirror wide space are shown below the 2SZ design and a 2 × 2 mirror grid with the 1 μm distance between mirrors is shown below the 4SZ design. Feature size is 56 × 56 μm for 2SZ and 28 × 28 μm for 4SZ. (**B**) Representative lithographic mask of the CB_120 library design containing ∼300 000 features 1 × 1 mirror large in a checkerboard pattern, as shown next to the bitmap mask. Feature size is 14 × 14 μm. (**C**) Overview of library synthesis by MAS and subsequent off-chip library preparation. In the 2SZ and 4SZ designs, 20 000 67mers containing fixed 5′ and 3′ poly-dC and poly-dA ends were synthesized, while the CB_120 library was composed of 300 000 120mers with the necessary sequencing adapters. All oligonucleotides were synthesized on a 3′ ester-modified dT unit (structure shown on the synthesized microarray, with red spheres representing base protecting groups). Deprotection and cleavage allows for the library to be recovered from the surface by applying a small amount of water, and the single-stranded oligonucleotides were then transformed into a dsDNA library with sequencing adapters using a commercially available kit, followed by PCR and Illumina sequencing.

All oligonucleotides in this study were synthesized by photolithography (Figure 1A) using phosphoramidite chemistry adapted to UV-mediated 5′-deblocking (Figure 1B), A library of 20 000 67-nt long oligonucleotides was designed so as to cover a large sequence space, with all combinations of 7-nt stretches present in 7 different oligomers. The initial array design consisted in randomly distributing the sequences over the synthesis area in features 4 × 4 mirrors (or pixels) in size and each separated by two mirrors (‘street size’ 2; 2SZ, Figure 2A). Each sequence was synthesized on a single feature. Sequencing the cleaved library (Figure 2C and Supplementary Figure S1 and S2, Supplementary data) revealed an average error rate of 6.3% per bp, certainly much higher than commercial array manufacturers, but in line with previous observations (26). The error-rate can be broken down into three components: a deletion rate calculated at 4.65% per bp, an insertion rate at 0.58% per bp and a substitution rate at 0.98% per bp (Table 1). From a synthetic point-of-view, a deletion corresponds to an incomplete removal of the photosensitive protecting group preventing the next coupling from happening. In the current MAS approach, we use the photolabile benzoyl-2-(2-nitrophenyl)propoxycarbonyl (BzNPPOC) as 5′-OH protection. We previously showed that photolysis of BzNPPOC proceeds twice as fast as regular NPPOC, requiring a radiant exposure of 3 J/cm^2^ to achieve ∼95% photodeprotection efficiency (32), which is the UV exposure parameter we selected. This means that ∼5% of BzNPPOC are not removed, making the next phosphoramidite coupling impossible and leading to a deletion. Thus, a 4.65% deletion rate is in total agreement with photolysis efficiency measured on-array using fluorescence labeling.

**Table 1.** Synthetic error-rate of DNA libraries sequenced in this study, reported as % per bp

Library	Average total error-rate (% per bp)	Deletion	Insertion	Substitution (all)	Substitution (G→T)	Deletion (x1)	Deletion (x2)	Deletion (x3)
*2SZ*	6.3	4.65	0.58	0.97	0.31	4	0.55	0.1
*4SZ*	5.9	5.04	0.17	0.56	0.22	4.3	0.62	0.12
*Capped 2SZ*	6.2	5	0.56	0.56	0.07	4.3	0.59	0.1
*CB_120*	21.8	13.6	4.6	3.6				

The insertion rate is the counterpart of incomplete BzNPPOC removal and results from the unintended exposure of surface features other than those being directly illuminated. At 0.6% per bp, it appears to be an unlikely event when features are separated by a ∼28 μm minimum distance (≡ 2 mirrors). This is particularly relevant to mention since photolysis follows first-order kinetics and there is substantial BzNPPOC removal even under short exposure to 365 nm UV light. Local flare, i.e. light scattered from the edges of mirrors (either turned on or turned off at any given exposure event) and imaged onto the slides, as well as diffracted light, would result in photodeprotection in the immediate proximity to the synthesis pixels, mostly corresponding to the ∼1 μm gap between adjacent mirrors. With the 2SZ design, potential sources of unintended photodeprotection are diffraction, local or global flare (originating in dust and imperfections of optical elements), but could also be the consequence of reflected UV light. Indeed, UV light, after exiting the second slide, reaches the quartz block on which the arrays are installed. At the glass/air and air/quartz interfaces, incident light can be reflected due to changes in refractive indices, about 4% of total light per interface. This is particularly problematic for light reflected at the second air/quartz interface. Since this surface is already about 11 mm from the array surface, the reflected light exposes a large surface on its return due to the 4.6° divergence of the beam in the 0.08 numerical aperture imaging system, resulting in reflected UV light exposing features much further away from the intended area. To counter this effect, we filled the back chamber of the synthesis cell with a solution of β-carotene in CH_2_Cl_2_ (31,38). The β-carotene effectively absorbs the UV light, thus preventing it from reflecting back as it exits the quartz window (Figure 2C). On top of the absorbing properties of β-carotene alone, the air has been replaced with dichloromethane, creating solvent/solid interfaces with a lower reflection coefficient (∼0.04%) due to the refractive index of dichloromethane (1.42) approaching that of glass and quartz. Reflections between the glass slides are effectively suppressed by the DMSO-based exposure solvent which has an index of refraction that closely matches that of the glass slides (1.48).

The substitution rate (0.97% per bp) catalogs all possible *M*→*N* nucleotide replacements (Supplementary Table S1, Supplementary data). The largest contributor to error in this case is G→T substitution, alone at 0.32% per bp, when all other *M*→*N* substitutions occur with a constant 0.04–0.07% error rate. We can explain this peculiarity by taking into consideration the notably lower coupling efficiency of G phosphoramidites relative to the other three (97–98% stepwise coupling efficiency (30,39)) and the cycling order of nucleotide coupling, always following A→C→G→T. Since we bypass capping, any failed coupling will likely be followed by a successful coupling with the next incoming phosphoramidite, a prime example of this situation being the G→T transition. However, the previously calculated coupling efficiency for G seems to be underestimated and would, based on substitution rate alone, be closer to 99.7% stepwise. Actually, all substitutions following the order of nucleotide coupling (A→C; C→G; G→T and T→A) inform on the corresponding coupling efficiency, which in this way appear to reach >99.9% for A, C and T. Introducing capping to the equation yielded a library of essentially similar quality (6.2% error rate) with marginally higher deletion rate (5.04%). The substitution rate however has been reduced by a factor of almost two, to 0.56% per bp, the largest reduction coming from the G→T substitution (from 0.32% down to 0.07%), highlighting the beneficial effect of capping unreacted hydroxyl groups after G couplings. The substitution rate is now within the range of all other *M*→*N* error-rates (0.04–0.07%) seemingly unaffected by capping, which either indicates that capping efficiency is as high as coupling efficiency (∼99.93%) or, concomitantly, that the technical limitations of sequencing have been reached. Since capping in our MAS system corresponds to coupling a DMTr-dT phosphoramidite to unreacted 5′ hydroxyls, capping efficiency approaches amidite coupling efficiency and will therefore only be as high as that of BzNPPOC-dT.

We then aimed at increasing the distance between features to see the effect on the insertion rate. Spacing the features with four mirrors (street size 4, SZ4, Figure 2A) logically shrinks the feature size to a 2 × 2 mirror grid in order to fit 20 000 unique sequences. Only about 2 million reads were obtained (compared to 4.2 million reads for 2SZ). The insertion rate fell from 0.58% to 0.17% per bp, due to scattered light statistically less likely to trigger unintended BzNPPOC removal on a surface where only 11% of the total area is being used for oligonucleotide synthesis (versus 44% for the 2SZ design). The deletion rate has marginally increased, to 5% per bp, which is important to note because light exposure is expected to vary as a function of feature size. In a 4 × 4 grid (2SZ design), central mirrors receive additional scattered light from neighbors, increasing photolysis efficiency in the grid's interior. This effect will be less pronounced in 2 × 2 grids where no feature is entirely surrounded by 8 similar spots (see Figure 2A), which could explain why the deletion rate is 0.4% higher under otherwise comparable synthesis conditions. Nevertheless, we cannot exclude an undetected slight drop in LED output. The substitution rate is lower than for the 2SZ design (0.56%) but is for the most part the result of G→T substitution, as expected for an uncapped library.

In all three library designs, the double deletion rate is also noticeable, at around 0.6% per bp and somewhat higher than what the theory would predict (0.16%). Three missing nucleotides, statistically at a 0.006% chance of happening, still occur at 0.1%. Multiple nucleotide insertions are, on the other hand, a negligible event.

### Synthetic and photochemical error rates: 30 0000-sequence library

**Figure 3.**
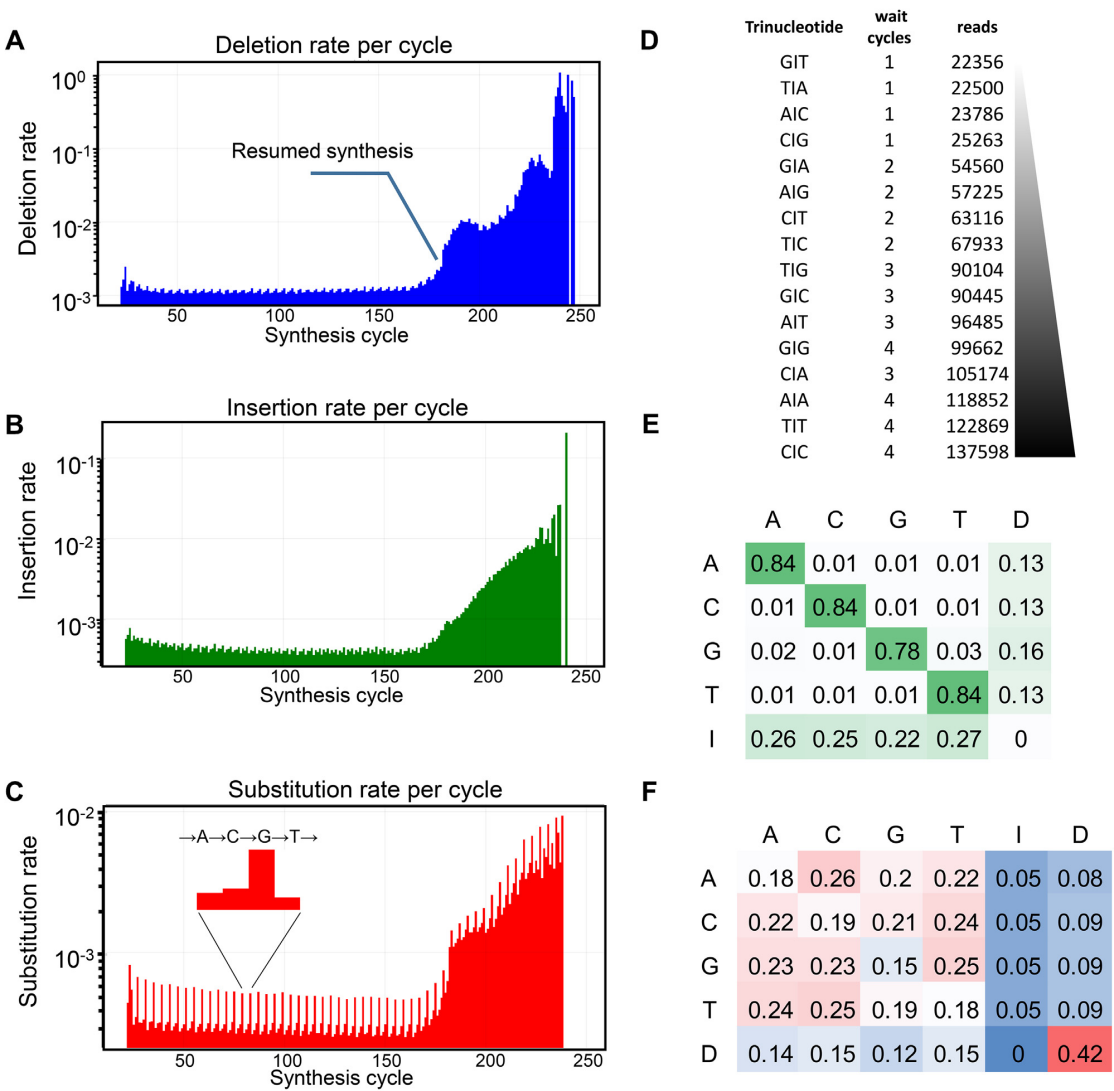
(**A**) Deletion rate as a function of synthesis cycle (15th to 250th cycle), previous cycles being the 3′ primer universally synthesized on all sequences. (**B**) Insertion rate as a function of synthesis cycle (15th to 250th). (**C**) Substitution rate as a function of synthesis cycle. The y axis is log scale, and ranges from 10^0^–10^−3^ for deletion rates to 10^−2^–10^−3^ for substitution rates. In the insert, the substitution rate attributed to phosphoramidite coupling, in MAS always following A→C→G→T. (**D**) Amount of sequences found with inserted nucleotides (I) and the corresponding number of cycles that had elapsed between the nucleotides 3′ and 5′ to the insertion. (**E**) Comparison of the chance (max: 1) to find nucleotide *Y* (column) when expecting nucleotide *X* (row). (**F**) Proportional error-rates as a function of base identity, I = insertion, D = deletion, A, C, G and T = substitution. Proportional error-rates add to 1 when summed up horizontally. The discontinuity in error rates corresponds to a library synthesis performed in two separate stages, the sharp change marking the point where synthesis was resumed.

In a next step, we increased the scale and density of DNA synthesis by MAS by preparing a library of ∼300 000 sequences each ∼120-nt long and already containing the necessary adapters for Illumina sequencing. This complex design requires half of all mirrors to be used for oligonucleotide synthesis, resulting in single-mirror feature size, each with one mirror of unused space in an overall checkerboard pattern (CB_120 design, Figure 2B). Illumina sequencing of the amplified library revealed a size distribution approaching the expected, adapter-free length (∼78-nt) with a maximum at the ∼70-nt mark. The library size after PCR was also found to match the expected length (Supplementary Figure S3 and S4, Supplementary data). From the 8.5 million reads, we evaluated the deletion rate at 13.6% per bp, with an average deletion length of 1.72 nt, an insertion rate at 4.6% per bp, and with an average insertion length of 1.44 nt (Table 1). While the error rate is much higher than expected in this particular case, a higher insertion rate is not surprising given not only the small gap between features but also the fact that each single-mirror feature can be unintentionally exposed to UV from all four corners. Since adjacent corner-to-corner distance is only about 1 μm, diffraction patterns in particular can contribute significantly to unintentional exposure in diagonally-adjacent pixels. However, upon closer inspection of the evolution of error rate as the synthesis progresses we remarked that the error rate (indels) remained fairly constant throughout the first 170 coupling cycles but soars in the last 90 cycles (Figure 3A and B). The discontinuity corresponds to resuming photolithographic synthesis as the integrated software of the automated synthesizer has a 250 base cycle limit, requiring an interruption in the synthesis for loading a second set of instruction files. Within the first 170 synthesis cycles, the average deletion rate actually fluctuates between 0.1 and 0.13% per cycle, then quickly increases to 1, then 10, then >10%. Similarly, the insertion rate remains consistently low during the first run, between 0.035 and 0.05% per cycle before progressively reaching 1% per cycle. Per bp, this corresponds to a 7.8–10% deletion rate and 2.7–3.9% insertion rate during the stable synthesis regime. Despite highly erroneous oligonucleotide synthesis and very dense use of the DMD capabilities, the original sequence information was retrieved with the help of retrieval algorithms.

We also studied the probability of insertions to occur during synthesis. We mapped the number of times an insertion was recorded to the number of synthesis cycles that elapsed between the nucleotides immediately 3′ and 5′ to the insertion. We found that insertions were more than five times more likely to happen if the 5′ nucleotide was coupled four cycles after the 3′ nucleotide than if they were coupled immediately one after another (Figure 3D). The wait number is incompressible as the MAS system follows the A→C→G→T coupling order, and a higher wait number corresponds to a higher number of exposure events taking place between the addition of the next nucleotide, thereby increasing the chance of an unintended photodeprotection happening in between. Next, we computed the match rate per nucleobase in Figure 3E. In this matrix, the match rate corresponds to the chance of finding a nucleotide *Y* (column) when expecting nucleotide *X* (row). We therefore read an 84% chance of finding an A when expecting A, and a ∼13% chance of finding a deletion. The match rate is noticeably lower for G (78%) and in parallel, the deletion rate for G appears higher (16%). This discrepancy can be understood in terms of G coupling efficiency. The G phosphoramidite has the lowest coupling efficiency of all four monomers, and any 5′-OH which failed to react with a G amidite will get the chance to couple with the next phosphoramidite since we do not cap after the coupling step, and the next incoming phosphoramidite is almost invariably T. However, because of the random generation of oligonucleotide sequences, statistically, 25% of all G are followed by a T. For those sequences, a missing G followed by T may be mistakenly interpreted as a deletion when it would simply be due to failed G coupling. This scenario explains the higher deletion rate apparently detected for G in Figure 3E. The last row of Figure 3E illustrates the proportional distribution of nucleotide insertion, and is quite expectedly the lowest for G insertion given its lower coupling efficiency. Finally, we looked at the distribution of 5′-*XY*-3′ dinucleotides (Figure 3F). The statistical distribution of the identity of two consecutive nucleotides is fairly balanced, as expected from random sequences, with interesting lows, particularly in the G and T columns. A lower proportion of *X*G relative to other *XY* dinucleotides (*X*: row, *Y*: column) can be explained by a lower coupling yield for the G phosphoramidite, indistinctly affecting any base to which G was supposed to couple. Homo-dinucleotides are less well represented, which can again be explained by incomplete coupling reactions: any failure of the first amidite in an *XX* system will create three different coupling opportunities before the second *X* amidite is incorporated. GG dinucleotides are the most severely affected due to the inherently lower coupling yield of G. Insertions are equally distributed amongst all four bases and so are deletions, indicating that nucleobase identity is irrelevant in the process of BzNPPOC removal, whether intentional or not, i.e. the photodeprotection quantum yield is nucleobase independent. However, a deletion is significantly more likely to be followed by a second deletion (42% of all possible cases of *DY*, D = deletion). Two consecutive deletions, even at 95% photodeprotection efficiency, are statistically unlikely to occur (0.05^2^) which hints at another mechanism being responsible for multiple consecutive deletions, possibly because of chemical degradation during synthesis due to phosphite triester hydrolysis or because of the presence of defective pixels within the DMD eliminating all UV exposure on the corresponding feature.

In all three plots of Figure 3, a pattern emerges where the deletion/insertion/substitution rate gradually increases and then returns to the lowest value, in a constant four-cycle rhythm. While insertion and deletion rates remain constant regardless of nucleobase, the substitution rate is very dependent on base-specific coupling efficiency, and thus the periodically appearing high substitution rate (Figure 3C) can be attributed to failed G couplings. The highest substitution rate always corresponds to the highest deletion rate and lowest insertion rate. Since the highest substitution corresponds to a failed G coupling and because the most likely substitution to occur is G→T due to the cycling order, it seems likely that for sequences containing a GT dinucleotide, a G→T substitution could have been considered a deletion. This would explain the variations, albeit fairly small, of deletion rate as a function of synthesis cycle.

### Error-rate as a function of the *xy*-position in the synthesis area

**Figure 4.**
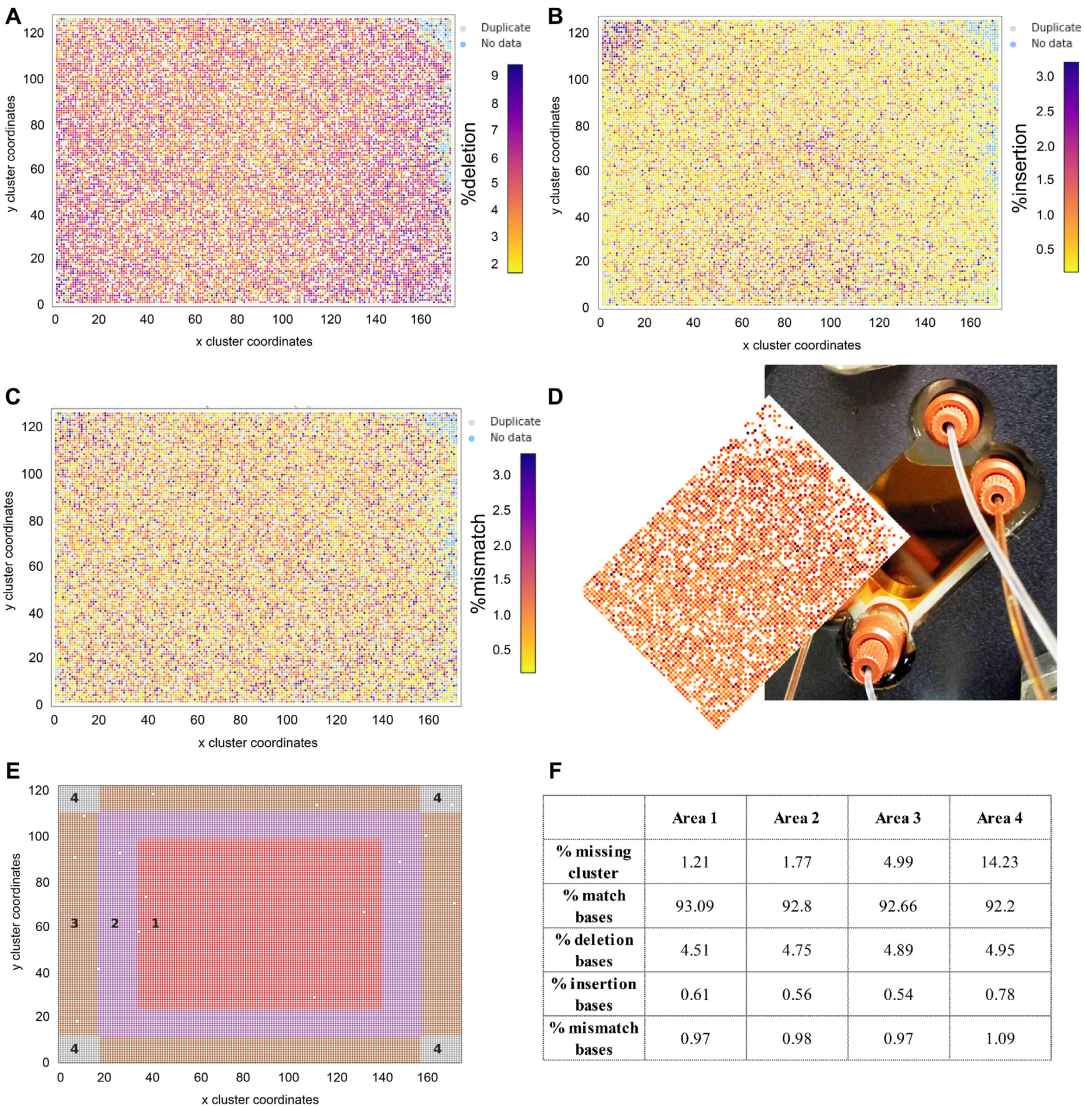
Distribution of error rate as a function of spatial coordinates on the 2SZ-design array. (**A**) Deletion rate; (**B**) Insertion rate; (**C**) substitution rate; (**D**) distributed error-rate in the synthesis area tilted 45° counterclockwise to illustrate the top-right corner of the array being unevenly covered by the β-carotene solution (orange) filling the back chamber of the synthesis cell; (**E**) synthesis layout subdivided in four areas and the associated error-rates (**F**). Coordinates are given as a function of cluster localization, given that each sequence is synthesized on features 4 × 4 mirrors large.

We graphed the average error-rate of each of the 20 000 sequence (2SZ and 4SZ) and 300 000 sequence library (CB_120) as a function of their origin in the synthesis area. The distribution, shown for the 2SZ design in Figure 4, reveals clear disparities in sequence fidelity as a function of spatial organization, particularly for deletion rates (Figure 4A). Lower deletion rates appear to be confined within a central area, indicating that the edges receive lower UV light exposure. This is most likely due to inefficiencies in the spatial homogenization of light illuminating the DMD. The homogenizer is a long rectangular mirrored tunnel with cross-sectional aspect ratio corresponding to that of the DMD. Light from the square-shaped UV LED emitting surface is focused into the homogenizer where the spatial emissivity pattern of the source is spatially homogenized and transformed to match the aspect ratio of the DMD through multiple reflections. Inefficiencies in this process, such as misalignment, insufficient mirror reflectivity or insufficient tunnel length, will predominantly lead to lower intensities at the corners and short edges of the DMD. Interestingly, the top right corner of the area contained ∼90 oligonucleotide sequences which were non-readable due to very high-error rates (Supplementary Table S2, Supplementary data). We noticed that this area perfectly matches the location of an air meniscus over the otherwise filled β-carotene back chamber. The reaction chamber being tilted 45° relative to the plane of the optical circuit, the cross section of the corner corresponds to the meniscus at the air/CH_2_Cl_2_ interface (Figure 4D). Non-readable sequences were also sparingly found at the very edges of the synthesis area, which again indicates a light intensity drop-off at the periphery of the DMD. High insertion rates (Figure 4B) are also heterogeneously distributed, appearing either within the central area where UV illumination is the strongest, but also at the top left corner. There, UV light reflection is prevented by a total coverage with β-carotene, so unintended photodeprotection must be the result of higher local scattering of light, for example, if a bubble is temporarily trapped in that corner. Substitution rates (Figure 4C) seem to be generally increasing towards the top left and bottom right corners. Since the presence of substitutions is independent of exposure to UV, a higher substitution rate would indicate lower coupling yield, which could be due to a slower reagent velocity away from the main bottom left→top right trajectory of fluid flow across the surface (28). Under very short coupling conditions (15 s), lower exposure to freshly activated phosphoramidite could have a noticeable effect on the coupling yield. We then looked at how the average error rate increases as we move away from the central area. To do so, we designated four subsections in a concentric rectangular pattern, with area 4 representing all four corners (Figure 4E). The deletion rate clearly increases from 4.5 to 5% as we move towards the edges, but insertion and substitution rates only worsen once reaching the corners of the DMD (Figure 4F). In addition, the insertion rate is higher in the central area (0.61% vs 0.55% in areas 2 and 3) as can also be observed in Figure 4B. The amount of missing clusters also increases towards the edges, which is in line with the assumption that light power decreases at the borders of the DMD due to limitations of the optical circuit.

The fact that sequences are undecipherable when MAS is being performed without the UV light absorbing solution is a testament to the impact of reflected light on synthesis fidelity, as unintended photodeprotection is going to affect many neighboring features simultaneously at each exposure event. This in turn is likely to yield sequences significantly longer than those designed, considering that the total number of synthesis cycles to make a library of 67-mers approaches 185. Spacing the features further apart, all the while reducing their respective size (4SZ), actually had the opposite effect on synthesis fidelity, particularly within the dimmer edges of the synthesis area where a significant proportion of sequences contained too many errors to be attributed (blue data points, Supplementary Figure S5C, Supplementary data). Based on the dynamic ranges of deletion and insertion rates in the 4SZ library, it appears to be due to a gradual increase in deletion rate as sequence position moves away from the center of the DMD and it explains the fewer numbers of reads by NGS. Similar analysis of the capped design (Supplementary Figure S6, Supplementary data) reveals lower deletion rate in the center of the array, which is accompanied with a higher insertion rate in that region too. As for the uncapped 2SZ design, the deletion rate increases from center to edges to corners while the insertion rate follows the opposite trend. In the 4SZ and capped 2SZ designs, we were able to prevent the formation of an air bubble, which explains why the top right section of the corresponding synthesis areas (Supplementary Figure S5 and S6) is noticeably better read.

**Figure 5.**
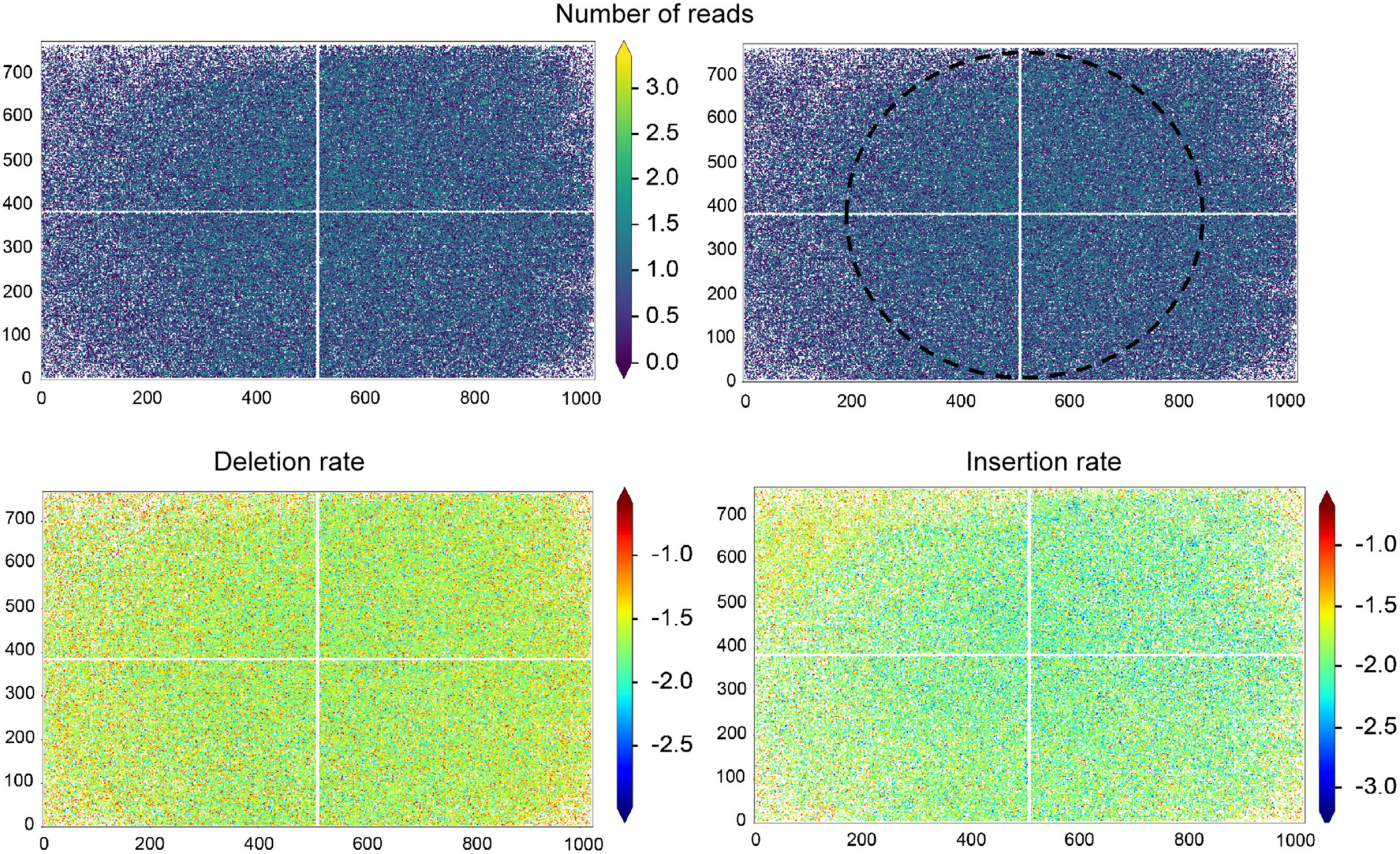
Spatial distribution (log scale) of the number of readable sequences and the indel rate as a function of array coordinates in the 300 000-sequence library (CB_120). Top right: a circle broadly delineates the area where sequences are synthesized at higher fidelity.

The denser CB_120 library, when plotted as *x*,*y* coordinates, also shows the same disparity in error rate, where a central disk occupying just over half of the total synthesis area not only averages lower deletion and insertion rates, but also a higher numbers of reads (Figure 5). The number of readable sequences quickly drops as the assigned feature coordinates move from the center of the DMD to the top left corner, which is also where a higher insertion rate is measured, approaching 2.5% per bp as a lower bound.

### Error-rate as a function of nucleotide position

**Figure 6.**
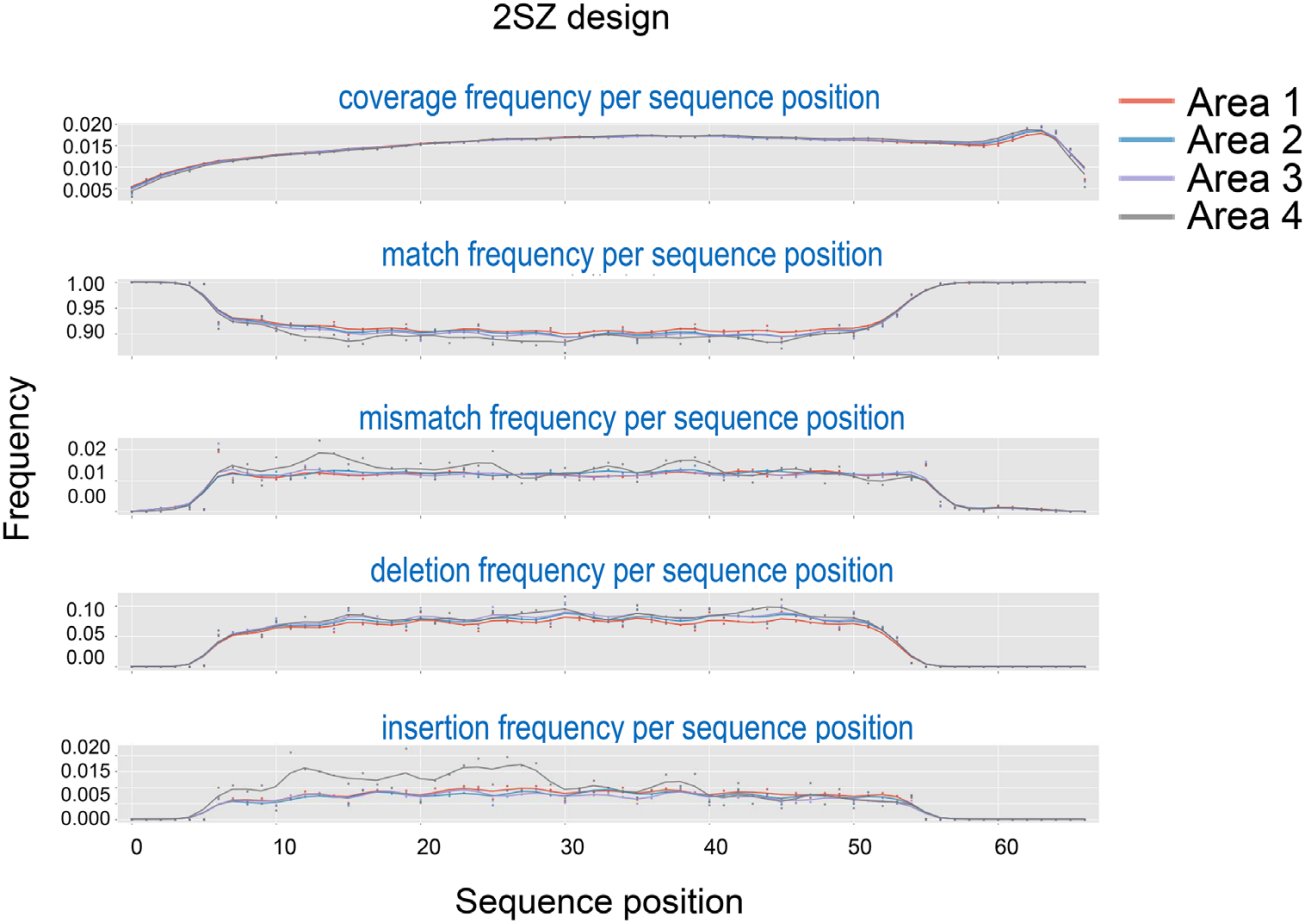
Match and error rate (% per position) as a function of nucleotide position for all 20 000 sequences in the 2SZ design, for all four areas. Position is shown in the 5′ to 3′ direction.

We next evaluated how the error rate varies relative to the extent of oligonucleotide synthesis and nucleotide position within the sequence (Figure 6 and Supplementary Figure S7 and S8, Supplementary data). The four concentric areas (1–4, center to corners same as Figure 4E) were individually investigated. In all designs, we see a clear dependence of the error rate with positioning; the 3′ and 5′ ends being essentially error-free, which correspond to homopolymeric regions synthesized at both extremities. This could be a consequence of lower synthesis error but it is more likely the result of alignment soft-clipping. The match rate stabilizes around 90–92% on average for a large central part of the sequence, area 1 (center) noticeably less error-prone than area 4 (corners). The deletion rate remains fairly constant in the middle section, between 7 and 8%. These values appear higher than the average deletion rate reported in Table 1, likely because of the homopolymeric regions common to all sequences. The insertion rate oscillates between 0.5 and 1% in the central section but, interestingly, moves closer to 1.5–2% in area 4 toward the 5′ end. We believe that this change in insertion rate is due to evaporation of the UV-absorbing fluid over the course of the synthesis, which eventually exposes the top right corner to reflections (see Figure 4D). With the chemical synthesis of DNA proceeding in the 3′→5′ direction, the 5′ end of oligonucleotides will be more affected by an increasingly imperfect β-carotene coverage. However, this increase is not a true quantification of the insertion rate at the exposed top-right corner since area 4 includes all four corners. Such an increase in insertion rate at the corners towards the end of the synthesis is not present in 4SZ and capped 2SZ designs (Supplementary Figure S7 and S8) since β-carotene coverage remained stable throughout the corresponding syntheses.

In both deletion and insertion rates, an odd pattern emerges whereby each fifth nucleotide displays unusually high deletion/low insertion rate. Since the sequences are random and are largely not synthesized at the same pace, it seems unlikely to be related to a periodically weaker exposure event, despite the correlation between high deletion and low insertion, and should not be due to a slower photolysis rate for a specific nucleotide, which should be manifest with a periodicity of 4, not 5. At first glance, one could see a side effect of the higher substitution rate whenever a G is coupling and how a G→T substitution can be mistakenly attributed to a G deletion, as was observed and explained in Figure 3D. A periodically higher substitution rate is absent from Figure 6 as the data is an aggregate of all 20 000 sequences. Introducing capping ensures that the G→T substitution is minimal (Table 1) but this extra step did not remove the deletion and insertion rates spikes (Supplementary Figure S8). Based on this observation, and taking into account the fact that all sequences are randomly generated and do not periodically show a greater or lower G content, we surmise that the periodicity of 5 could be an artefact of sequence alignment.

## DISCUSSION

The synthesis of oligonucleotides by photolithography is a process that integrates photochemistry into conventional, cycle-based, automated nucleotide coupling. While the formation of a phosphorus bond, the potential side reactions, the protection and deprotection stages are very well understood and applicable to high-throughput array synthesis, the light component in oligonucleotide elongation is the domain of MAS, which is far from being a ubiquitous piece of instrumentation. MAS is a complex approach to DNA synthesis, where photochemistry is designed to take place simultaneously at precise locations on a small surface area all the while controlling potential adverse effects related to light and optics. We have previously identified which optical errors will affect synthesis efficiency and fidelity (30): diffraction of light and scattering from the edges of a mirror will expose each synthesis pixel perimeter to UV while global scattering randomly expose the entire surface. In addition, the choice for an optimal photosensitive protecting group, as well as the mechanistic aspects of *o*-nitrophenyl photocleavage, is key to the success of photochemical oligonucleotide synthesis and, unsurprisingly, they have attracted considerable attention (40–47). We became invested in improving the photolysis efficiency using new *o*-nitrophenyl derivatives for DNA photolithography (32). But while simulations and on-array experiments, primarily hybridization and terminal fluorescence labeling, have allowed us to understand which parameters govern the quality of DNA synthesis by MAS, off-array validation of those observations via sequencing was still missing. This is particularly important in order to secure reliable protocols for the preparation of very complex libraries, when MAS is being used at its full scale and potential, since sequencing can reveal unexpected sources of error as well as provide a more quantitative and detailed understanding of synthesis accuracy.

Errors introduced during the preparation of synthesized DNA libraries are a known aspect of oligonucleotide and microarray chemistry. To alleviate the issue of imperfect synthesis, significant effort is currently dedicated to building a technical coding and decoding framework which can reliably recover DNA-based information from highly erroneous source material, either due to degradation or to synthesis errors (48,49). For digital data storage purposes, it is likely that the burden will be carried by error correction, as computing power is considerably cheaper than engineering an optimal and near 100% perfect chemistry and since DNA naturally degrades over time, even under the most safeguarded conditions (1). We recently showed how a high error-rate regime in array photolithography could still yield readable libraries given the proper correcting algorithms (26). Powerful code repair is but one of the reasons why our synthesis parameters do not aim to achieve >99% photodeprotection efficiency, i.e. the lowest deletion rate. Convenience and throughput play major roles as well, as increasing radiant exposure from 3 to 6 J/cm^2^ results in a doubling of the exposure time which, logically, almost doubles the total synthesis time. But while it may be important to achieve the highest possible synthesis fidelity in DNA libraries for gene assembly purposes, a 4–5% increase in photolysis efficiency will almost always mean a large increase in insertion rate due to the now longer time during which the entire surface is exposed to globally scattered light. Because the photolysis rate follows first order kinetics, unintended photolysis (resulting in insertion errors) will quickly rise for a small marginal gain in deliberate photodeprotection. Since the slope of the photodeprotection curve is about 800 times steeper at low exposure than the slope between 95 and 99.7% photolysis (3 and 6 J/cm^2^ exposures of BzNPPOC), small improvements in the deletion rate will result in large increases in the insertion rate, unless the optical contrast can be improved. Optical contrast in this context is the light exposure ratio between intentionally exposed and unexposed pixels. Diffraction and local scattering contributions to loss of contrast can be greatly reduced with a checkerboard layout, where adjacent pixels used for synthesis are only cross-contaminated in their corners, or mostly eliminated by using single pixel borders around each synthesis feature. The contribution of global scattering to reduced optical contrast can also be minimized with less dense layout, as global scatter is proportional to the total amount of light directed to the surface. Of course, all of these approaches result in a fewer synthesizable sequences and/or a smaller yield of each oligonucleotide; therefore, when large numbers of sequences are needed and when computational or experimental approaches to compensate for errors are available, it can make sense to synthesize at high error rates.

All DNA libraries, regardless of density and pattern, were synthesized according to the same chemical and exposure protocols. The denser design, CB_120, counting 300 000 sequences, has no features adjacent side-to-side, but are arranged in checkerboard-type pattern. It is not surprising to measure the highest insertion rate in these libraries, between 2.7-4.6% per bp, considering that the 0.8 μm gap between mirrors is less than the 2.4 μm diffraction-limited resolution of our 0.08 numerical aperture (*NA*) MAS. This resolution of the imaging system, based on the Rayleigh criterion (*R* ≈ *λ*/2*NA*), was chosen as an optimal compromise between system cost, resolution and scattering tolerance (30). Higher resolution increases the size, cost and complexity of optical systems; and while better resolution decreases diffraction-based light crosstalk between immediately adjacent synthesis pixels, it also results in increased sensitivity to global scatter. Whereas resolution improves linearly with increasing *NA*, the amount of scattered light that can reach the array increases as the square of the *NA* (30). Resolution can also be improved by reducing the wavelength of light, but sources below ∼320 nm are likely to cause photodamage to the DNA, and intermediate sources would provide only a marginal improvement in resolution

Light diffraction from mirror edges results in a pattern of fringes that extend into neighboring pixels. The resulting unintended exposure decreases quickly with distance, to about a 10th of the intensity of the deliberately exposed pixel within 0.8 μm (the gap distance) and decreasing sub-exponentially in a Fresnel diffraction pattern (50). The cumulative unintended exposure in each adjacent pixel sharing a common side reaches about 0.5%, and in adjacent pixels sharing only a corner, likely less than about 0.05% (30). Scattering from the edges of mirrors is imaged onto the synthesis surface and therefore this source of unintended exposure is mostly confined to the gap regions. This edge scatter is independent of whether the particular mirrors are directing light towards the array or not and therefore accumulates even during exposure cycles when the relevant pixels are not being directly exposed. Diffraction and edge scattering are too small to explain the overall >2% insertion rate in the 300 000-sequence library and a large contributor to it must come from global scattering. Such scattered light is not imaged onto the synthesis surface, but is distributed essentially randomly. The amount of global scatter is proportional to the amount of light directed to the surface, and hence to the number of mirrors used for synthesis. Indeed, in the case of the 20 000 sequence libraries, having at least a two-mirror distance between features brings the insertion rate down to 0.58% per bp, and further distancing to four mirrors brings the insertion rate to 0.17%. This reduction is close to the factor of four expected reduction in unintended exposure achieved by reducing the active mirror density from 44 to 11% in these two libraries, and confirming that a margin around features is sufficient to eliminate most contributions to insertions by diffraction and local scatter. Thus, reductions in density reduce the insertion rate not directly because of the increased distance between features, but because less total light is directed towards the surface in any given exposure, proportionally reducing global scattering. Based on the insertion rates for the 20000-sequence library synthesized using 44% of the mirrors, the 300 000-sequence library with a similar 50% mirror density should have an insertion rate of ∼0.68% based on global scattering alone. From this we can estimate that diffraction and local scattering account for a 2–3% insertion error rate in the checkerboard layout. Full density arrays aiming at preparing up to 786 432 unique sequences will have very high insertion rates since all sources of unintended exposure will contribute maximally.

Another result from the sequencing data is that reflections from light exiting the flow cell must be efficiently suppressed. Such reflections are analogous to local scattering and diffraction in that their impact on error rates is spatially defined by the pattern of mirrors and the optical path within the flow cell. The reflection from any exposed feature will expose a larger surrounding area due to the divergence of the beam of light. We can suppress these reflections by introducing an absorbing solution of β-carotene in dichloromethane into the 250 μm gap between the slide and quartz block. The absence of the solution behind a small corner resulted in the synthesis of unsequenceable DNA in this location.

The mechanism of deletion is simply related to photodeprotection efficiency, and we have shown how our on-array measurements of BzNPPOC removal, using either hybridization or terminal labeling with a fluorescent phosphoramidite, match the sequencing data presented here. But the sequencing data is more sensitive and reveals a more complex picture, a spatial dependence of synthesis fidelity. The central part of the array is generally less error-prone indicating inefficiencies in the light homogenization that results in less light reaching the edges of the synthesis area. Such spatial patterns in illumination efficiency can be addressed with better engineered light homogenizers, or alternatively, by exposing some areas of the surface for longer, by digitally modulating the time each mirror is in its ON position according to the measured pattern of underexposure.

The substitution rate is the only error type originating from phosphoramidite chemistry alone and, as expected, is most noticeable for G which couples less efficiently than A, C and T. Substitutions are not random and can be traced to the next incoming phosphoramidite due to the invariable cycling order. While G→T substitutions can be misinterpreted as G deletion, a simple solution is the introduction of a capping event after each amidite coupling, reducing the G→T substitution rate from 0.3% to 0.07% per bp, but a longer G coupling time or an increased concentration of amidite in acetonitrile from 0.03 to 0.05 M should be just as effective.

Performing library synthesis in a single run appears to be a pre-requisite to higher oligonucleotide quality, since at the software and hardware levels, complex designs currently force our fabrication protocols to separate the run into two parts but without having to disassemble the synthesis cell, which should therefore not change the alignment of the slide relative to the DMD. There is, however, a copious wash and drying event at the end of each run, which could contribute to changing the wettability of the surface and, in particular, lead to coupling and photodeprotection reactions being less effective. Removing the terminal wash should alleviate the issue or, alternatively, transforming each A→C→G→T cycle into an X cycle aggregating the four consecutive couplings into a single letter will allow for the 255-character limit to be overcome.

The sequencing analysis of high-density DNA libraries carried out in this study is valuable in several respects. It validates previous observations and measurements made on surface-bound oligonucleotides, and indicates that the process of cleavage, retrieval and amplification does not alter the outcome of error distribution. It is also, to the best of our knowledge, the first direct investigation and quantification of all possible sources of error in the synthesis of complex microarrays, and certainly so for light-directed array fabrication. The manufacture of oligonucleotide microchips remains extremely relevant in the context of novel applications requiring large quantities of nucleic acid material, and all approaches rely on conventional phosphoramidite chemistry. And whether light-directed or acid-mediated, *in situ* methods offer total control over the position of each sequence on the surface. The work undertaken here shows that sequencing synthetic libraries can return useful information about the various chemical aspects of oligonucleotide elongation but importantly, can also shed light on how error rates are unevenly distributed across the surface of the array. This reliable methodological pipeline we have assembled is therefore expected to be applicable to studying synthesis accuracy in inkjet and electrochemical array systems as well. Indeed, we present how single-stranded DNA, synthesized with or without primers and where each base has a >7% chance of being inaccurate, can be amplified and read, with the original sequence correctly retrieved. Shorter oligonucleotides resulting from incomplete photolysis are likely to have received the proper sequencing adapters since they are terminated with the same 5′ and 3′ functionality as for complete sequences. Only very short oligonucleotides resulting from chemical degradation were probably absent from the pool of amplified sequences, but the process of degradation would have affected all oligos in a sequence-independent manner. The process is scalable and was demonstrated to function with 50% array density, which should allow for full-density array synthesis to still produce sequenceable oligonucleotides despite the expected increase in insertion rate. In terms of scale however, the protocols used in this study for the preparation of libraries by MAS are far from being optimal: BzNPPOC protecting groups may be replaced by the SPhNPPOC, with a six-fold increase in photolytic efficiency. Next-generation DMDs (1080p or 4K resolution) would represent a significant improvement in throughput, allowing for 2 and 8 million features to be accessible within a similar area of a standard microscope slide. Finally, a deep understanding of the origin and extent of sequence errors in DNA array synthesis will be useful towards experiments based on the photolithographic synthesis of RNA and chemically-modified nucleic acid libraries.

## DATA AVAILABILITY

The panel design and sequencing analysis repository is available on Github (https://github.com/a-slide/DNA_photolitography_seq) and archived at Zenodo (https://zenodo.org/record/4524788).

Sequencing data for 2SZ, 4SZ, Capped 2SZ libraries were deposited at ENA under the following project id: PRJEB43002 (https://www.ebi.ac.uk/ena/browser/view/PRJEB43002).

## Supplementary Material

gkab505_Supplemental_File
