# Supplementary material for: Chemical and photochemical error rates in light-directed synthesis of complex DNA libraries
Source: Nucleic Acids Res. 2021 Jun 22;49(12):6687–701. doi: 10.1093/nar/gkab505 (PMC8266620; doi:10.1093/nar/gkab505)
Supplement: gkab505_Supplemental_File [file gkab505_supplemental_file.pdf]

SUPPLEMENTARY INFORMATION

Chemical and photochemical error rates in light-directed synthesis of complex DNA libraries

Jory Lietard[1,*], Adrien Leger[2], Yaniv Erlich[3], Norah Sadowski[4], Winston Timp[4,5] and Mark M. Somoza[1,6,7]

[1] Institute of Inorganic Chemistry, University of Vienna, Althanstraße 14, 1090 Vienna, Austria
[2] European Molecular Biology Laboratory, European Bioinformatics Institute, Wellcome Genome Campus, Hinxton, Cambridge, UK
[3] Erlich Lab LLC, Raanana, Israel
[4] Johns Hopkins University, Department of Molecular Biology and Genetics, Baltimore, MD, USA
[5] Johns Hopkins University, Departments of Biomedical Engineering, Molecular Biology and Genetics and Medicine, Division of Infectious Disease, Baltimore, MD, USA
[6] Chair of Food Chemistry and Molecular Sensory Science, Technical University of Munich, Lise-Meitner-Straße 34, 85354 Freising, Germany
[7] Leibniz-Institute for Food Systems Biology at the Technical University of Munich, Lise-Meitner-Straße 34, 85354 Freising, Germany

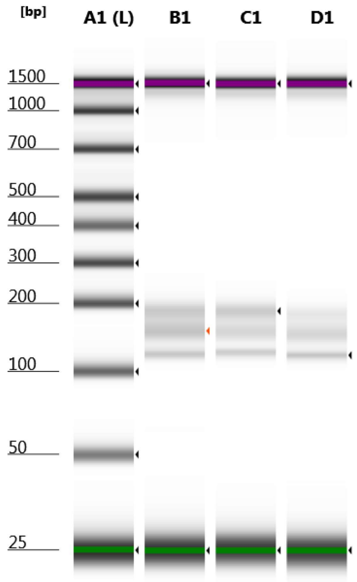

Figure S1. Electrophoretic separation of prepared libraries prior to sequencing using the TapeStation (Agilent) instrument and D1000 reagents. Lane assignments are as follows: A1 = Ladder, B1 = 2SZ, C1 = capped 2SZ, D1 = 4SZ

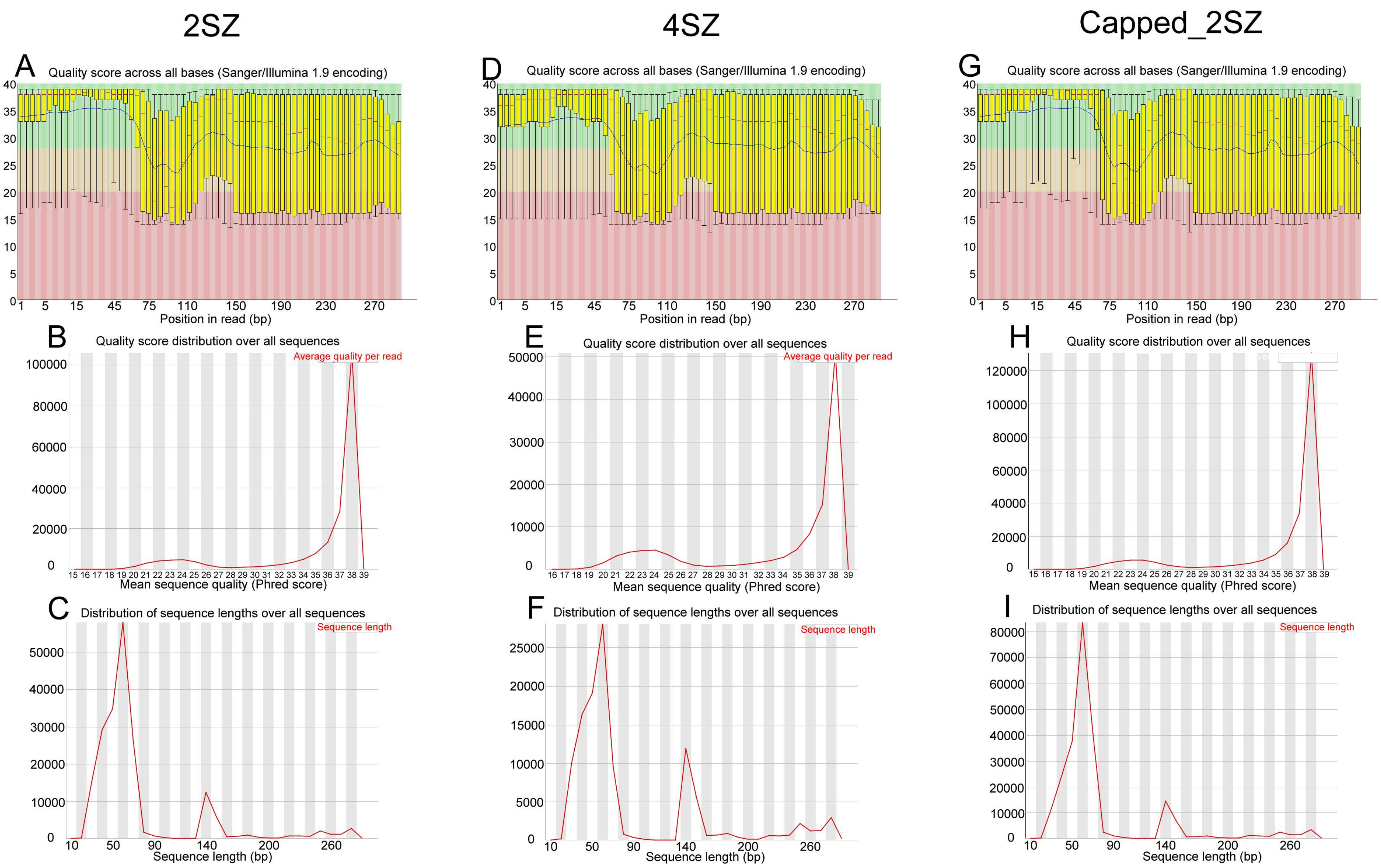
2SZ
4SZ
Capped_2SZ
A
Quality score across all bases (Sanger/Illumina 1.9 encoding)
Position in read (bp)
B
Quality score distribution over all sequences
Average quality per read
Mean sequence quality (Phred score)
C
Distribution of sequence lengths over all sequences
Sequence length
Sequence length (bp)
D
Quality score across all bases (Sanger/Illumina 1.9 encoding)
Position in read (bp)
E
Quality score distribution over all sequences
Average quality per read
Mean sequence quality (Phred score)
F
Distribution of sequence lengths over all sequences
Sequence length
Sequence length (bp)
G
Quality score across all bases (Sanger/Illumina 1.9 encoding)
Position in read (bp)
H
Quality score distribution over all sequences
Mean sequence quality (Phred score)
I
Distribution of sequence lengths over all sequences
Sequence length
Sequence length (bp)

Figure S2. Sequencing reads by Illumina HTS for the 2SZ, 4SZ and Capped_2SZ libraries, A, D, G: quality scores across all bases. B, E, H: quality score distribution for all sequences. C, F, I: sequence length distribution.

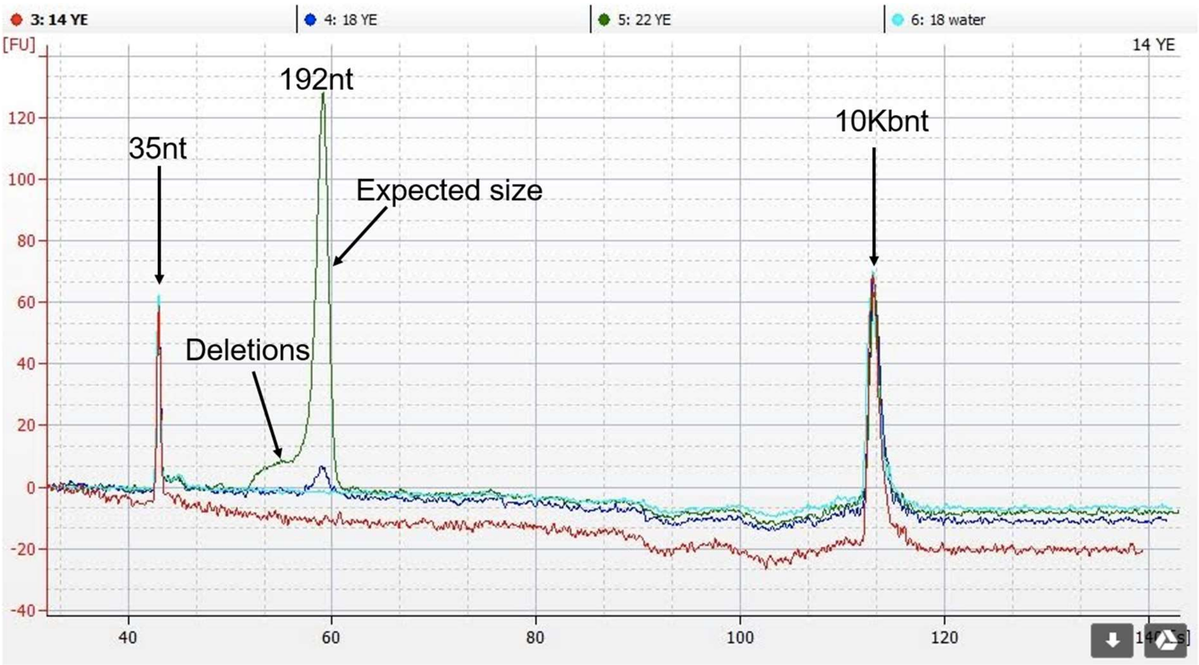

Figure S3. Bioanalyzer trace of the CB_120 library after library preparation and before sequencing. Traces were recorded after either 14 (red), 18 (blue) or 22 (green) PCR cycles, as well as for a water control (cyan). After 22 PCR cycles, the expected library size (192-nt) becomes visible.

Table S 1. Match rate and error rates measured for the 20000-sequence libraries (4SZ, 2SZ and capped 2SZ), arranged in descending order, in % per base pair. Single and multiple deletion and insertion rates are quantified and shown in the form "deletion: X" and "insertion: X" (X being the number of deletions). All types of *M→N* substitution rates are also calculated and given in the form "substitution: M>N" (N being found where M was expected).

type	Error-rate (%)			Reads		
	4SZ	2SZ	Capped 2SZ	4SZ	2SZ	Capped 2SZ
match	94,07	93,69	93,83	1992051	4270115	6461636
deletion: 1	4,40	4,06	4,30	93140	185045	296404
deletion: 2	0,62	0,55	0,59	13190	24933	40525
substitution: G>T	0,22	0,32	0,07	4735	14425	4539
insertion: 1	0,17	0,58	0,56	3525	26263	38801
deletion: 3	0,12	0,10	0,10	2540	4446	6988
substitution: A>C	0,05	0,08	0,06	1137	3636	4044
substitution: C>A	0,05	0,07	0,04	1084	3323	2924
substitution: G>A	0,04	0,07	0,09	856	3175	6158
substitution: C>G	0,04	0,13	0,05	766	5916	3111
substitution: T>A	0,04	0,07	0,05	763	3070	3434
deletion: 4	0,03	0,02	0,02	661	986	1524
substitution: G>C	0,03	0,04	0,05	615	1924	3131
substitution: T>G	0,02	0,05	0,03	510	2504	2179
substitution: T>C	0,02	0,04	0,04	476	1786	2709
substitution: C>T	0,02	0,04	0,04	458	2021	2622
substitution: A>G	0,02	0,04	0,03	365	1641	2116
substitution: A>T	0,01	0,03	0,03	314	1469	2209
deletion: 5	0,01	0,01	0,01	213	331	565
deletion: 6	0,00	0,00	0,00	78	165	287
deletion: 7	0,00	0,00	0,00	50	80	178
deletion: 8	0,00	0,00	0,00	25	55	75
insertion: 2	0,00	0,00	0,00	14	223	247
deletion: 9	0,00	0,00	0,00	14	32	57
deletion: 11	0,00	0,00	0,00	7	15	13
deletion: 10	0,00	0,00	0,00	6	13	26
insertion: 7	0,00	0,00	0,00	4	2	0
insertion: 11	0,00	0,00	0,00	2	0	0
deletion: 13	0,00	0,00	0,00	2	0	1
insertion: 5	0,00	0,00	0,00	1	0	0
deletion: 12	0,00	0,00	0,00	0	3	3
deletion: 14	0,00	0,00	0,00	0	0	3
insertion:10	0,00	0,00	0,00	0	0	2

insertion: 13	0,00	0,00	0,00	0	2	0
insertion: 3	0,00	0,00	0,00	0	4	9
insertion: 4	0,00	0,00	0,00	0	8	0
insertion: 6	0,00	0,00	0,00	0	2	1
insertion: 8	0,00	0,00	0,00	0	4	0

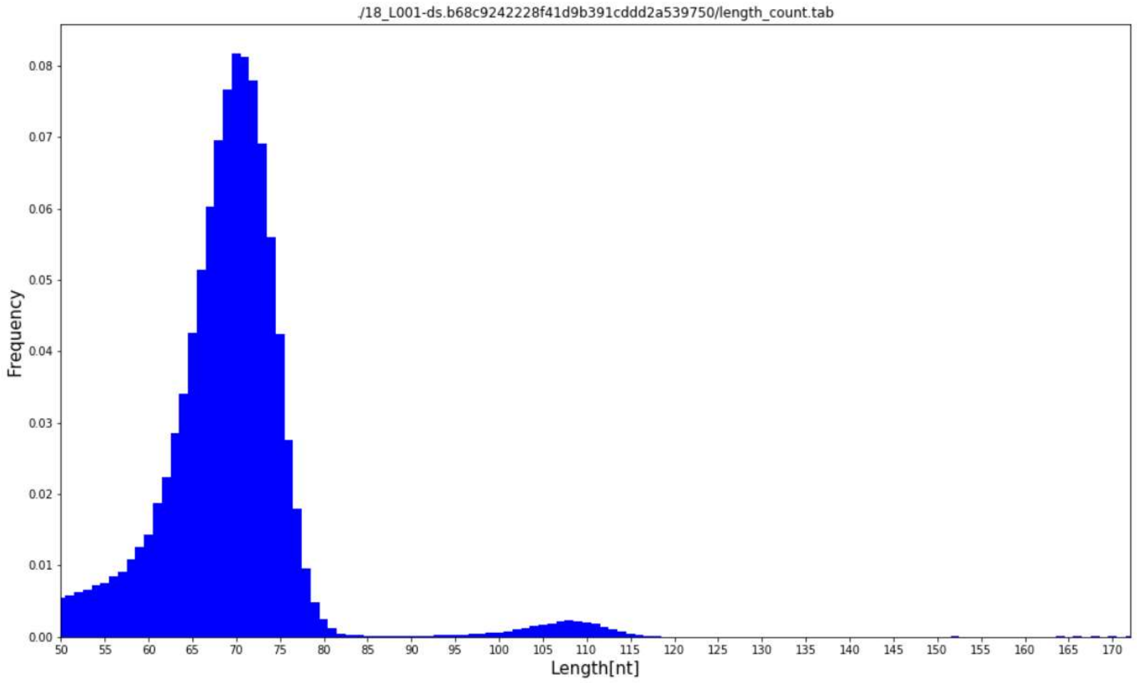

Figure S4. Distribution of sequence lengths after Illumina sequencing of the CB_120 library after read stitching (merging paired end reads into one long read by aligning the two ends against each other. We used PEAR (https://cme.h-its.org/exelixis/web/software/pear/doc.html) for this procedure). Oligonucleotides were synthesized as 120-mers, including Illumina adapters. The adapter-free portion of all oligonucleotides is ~78-nt long.

Table S2. List of oligonucleotide sequences irretrievable by sequencing and assigned to the top left corner of the synthesis area, where β-carotene imperfectly covers the backside of the synthesis chamber. X and Y feature coordinates are indicated next to the sequence.

Sequence (5′ to 3′)	X	Y
CCCCCCAACCTACCTATGGACGGTTATGTGTCGTGACTAGCATTCGTGTTCATGGCAAAAAAAAAAA	167	114
CCCCCCGGCGGTTCGACGTCGTATACATCTCACCAGCAGTTCAGTTATGTCTGCCGAAAAAAAAAAA	170	120
CCCCCCTAGGGAATTGTATGATGCTTAGCCGATCGATCATACTCGTGGCGCTACTGAAAAAAAAAAA	160	119
CCCCCCTCCTTCTCAACGACCAGTCGCGCTACCTTCGCCCGTGCTACCTTCGTCATAAAAAAAAAAA	160	123
CCCCCCTGTTCATACGTTTAATCGATATGGCGCGCGTAAACATTGAGCACACCCGGAAAAAAAAAAA	159	117
CCCCCCGTCACAAGGGTCGAAGAAATGGTTCTGTAGCGGTTATGTTACAATCTAATAAAAAAAAAAA	161	118
CCCCCCAGGGGACATCTTCATGGATGAGCCTGGAAATCCATGTGAAGAGTAGATATAAAAAAAAAAA	169	114
CCCCCCGCATATCCATCTTATAGTCATAGAAGCGAATGCCTCTAGCGGGGTGTTGCAAAAAAAAAAA	165	121
CCCCCCGGGAAGCGAACCGCCGACCACTGAAGTACTGTTATCCGGAGTGCATGTTGAAAAAAAAAAA	167	124
CCCCCCGTTGTAAACTATCAACGAGGTAGCGTGCGACACTACGATTGAAATGAGAGAAAAAAAAAAA	170	121
CCCCCCGCTCCGTCTTGGGAACTGTGATTGGTTAGTAGCGTGCGGTACTCATGTAGAAAAAAAAAAA	163	116
CCCCCCAACGGCAAAAGGAAACAAGCAGACCGTAAAGATTACTAGAGGAGGCGATGAAAAAAAAAAA	159	124
CCCCCCAGCGCTGGAGCATGCGGGCATTAGTGGTTCTTTTTACTACAGTCCTTCGGAAAAAAAAAAA	159	125
CCCCCCTTCCGTAGATCGTGAGATCATCATATGGTGTCGCATTTAGATCAATAGGCAAAAAAAAAAA	157	125
CCCCCCGTATGACTACGGGCAGCTGCGAGAACGCACGCTCTGCGTTCGTAACAATCAAAAAAAAAAA	158	126
CCCCCCAACTGCGACATGAGACTGCGTAATAGTCGATGCCCTTACGCCAATAAATGAAAAAAAAAAA	161	123
CCCCCCGATTCGTAGGCCGTGCCTCAGTCCACCGATCGCCTGGGCTGTGCTAACAGAAAAAAAAAAA	166	120
CCCCCCGGGCTAGCGTGTGGAGAAAACTTCATAATGTTATTGCGTCTGGTGTATTTAAAAAAAAAAA	169	111
CCCCCCGTGGATCATTCCGCTCCGGACGCATAGTCCTTATCTGCATATCGGCAATTAAAAAAAAAAA	157	127
CCCCCCGTAGACGCCCTTGATGTCGAGTACCGTTGCAGACCAAAGTCGGGCAAGGGAAAAAAAAAAA	170	125
CCCCCCGTGGTATTGTAACCAGTACACAGTGACTGACGCCTCTCATCGATGTGAACAAAAAAAAAAA	164	115
CCCCCCTCTCAGGCTGTATGTCGTAGTAGCTGCGGTCACATCCGGCGGAAGCCGATAAAAAAAAAAA	168	117
CCCCCCACGTTACCGACTGCCATGGCAGATGATGATACCGGTGTAATGCCCTTTGGAAAAAAAAAAA	160	116
CCCCCCTGGACGTGTCACCCAGTGGCTTCTCGTATATTCGATCCTATCGGATAAATAAAAAAAAAAA	164	117
CCCCCCTAGCCTAGGCAAGACTTTCACGCATAGCGTCTAGAGCCCCGGAAGCCCAGAAAAAAAAAAA	162	125
CCCCCCACCCACTGTTGAGAGCGTTCGACGCTGCCTATTGCGGTAGCCGTGAGAATAAAAAAAAAAA	167	121
CCCCCCTTAAGCAACGAACGGTGCGCGAGTATCGCCACTGAGTAGAGTAAGATATCAAAAAAAAAAA	169	116
CCCCCCTAATCATTCATAGGGCTAAGCCCTAGATTACGGGTACTTGACATTGCACGAAAAAAAAAAA	159	122
CCCCCCTAGGCTGAGAGATGCGGTCCGGGTTACATCATCGCGTAAACGCTGAGAGTAAAAAAAAAAA	164	120
CCCCCCTACAATGATCGTGACTTCCGAGCTGTGATTAGCAGTGTTAGCAACGCAGGAAAAAAAAAAA	169	117
CCCCCCGTTACATGAAGCTGAGTCCGAAGCAGTAAACCGTCGCACGTCATAGCACTAAAAAAAAAAA	168	123
CCCCCCAGCTGCAACAGAAACGGATGCGCACTACTGAGATCGCCGGTACTGTGTAGAAAAAAAAAAA	162	126
CCCCCCTGCCGATGGCAAGGGAGAATGGACTGGTATCTGTCTTGTTAGGAACGTGCAAAAAAAAAAA	156	127
CCCCCCAACGTAAAGACGTCCATCCTCCTCTGTCTACCCCTAAAAATATTCTGATTAAAAAAAAAAA	161	125
CCCCCCAACGGATGTAGATTTAACAGAAACTCACGAGACAACGGTCTTACCAGCAGAAAAAAAAAAA	164	127
CCCCCCTAAAATCCCTACGAACACGACGCTTCGAATGGGCCATCCGTGTCTATACCAAAAAAAAAAA	167	123
CCCCCCATGGTGTGATAACCCAACAGAGTAGCCGTATCCGCGCTGGTGAGAGTTCCAAAAAAAAAAA	167	116
CCCCCCACTGCAGAAGATACTCAAAGTCAACGATCGCGCCGCGTAGCGCGATACGCAAAAAAAAAAA	165	127
CCCCCCAGCAATACTTGTGCATCCACTGGACGTGAGAGTACTGGGTATGATCGGTTAAAAAAAAAAA	162	123
CCCCCCTGTCGTGTTATGAACGGCTATCCAGTATTGCGACACACGCGTATCGCAGGAAAAAAAAAAA	170	123
CCCCCCTCAGACGGGACGGGTGTTCTCAGTTGAAGCAGTCTGTAGGTTAGCAATACAAAAAAAAAAA	153	120
CCCCCCTATGCGACTGTCTCCAGGTGACGGGAGGGCGCACATTCGGTCTCACTTGGAAAAAAAAAAA	169	125
CCCCCCAGAGGAGATAGGCTGCGTAAGATTACGTGGCCCACGCGTATTGGATTCCGAAAAAAAAAAA	163	118

CCCCCCTTTTTCAGGTCATTCGCCTAGGGGTCTATACCGGCAGGGTGTCGAGGGTGAAAAAAAAAAA	167	118
CCCCCCTTGCTATGGAGCCTGACTGGTCCGGATAGTCTTTGCGGCTAATTGCTTATAAAAAAAAAAA	166	124
CCCCCCGACTACTCTCTAGCCGTCACGCGCACTTATCTATATTAGGCGTAACGTCTAAAAAAAAAAA	157	120
CCCCCCATCATATCACAGTAGTCGAGTGTCCTGATCTATAGATCTCTTATCAGGCGAAAAAAAAAAA	168	119
CCCCCCGTCTCGTTGTCCGCGCACCTGGGCCAGTTTGGTCACCGCCTTGGGCCCTGAAAAAAAAAAA	161	121
CCCCCCAACAAGGCCTCCCCGCTACTATGGATCAACACAGATCTATGCCTGCGCCGAAAAAAAAAAA	161	122
CCCCCCAGGACACAGCGCTGACTCGATTAGCTTTATCGATTCTGTTACTGGCCGTTAAAAAAAAAAA	170	116
CCCCCCATGAGAAACTGCACTGCAAGTGGACGCGGACGCGGAGGTATAGGCAAAGCAAAAAAAAAAA	169	124
CCCCCCTTCTCTACGGACAACGTCAAGGTAAGGTTGCCAGACACCACTGTTGATCGAAAAAAAAAAA	166	122
CCCCCCGTAGGTGCTTAGGCCGATAGCACACACCATGTTGAGGGACTTTCACGCGCAAAAAAAAAAA	168	125
CCCCCCGGTTTGGGTGACCCTGGTCACATCCGAACGCTTCCTGTTACGTGAGTGCTAAAAAAAAAAA	158	120
CCCCCCGTATCACTTGCCATAGAAACTGAGCTGCATGGGAGAAAAGCTTCATACAGAAAAAAAAAAA	162	122
CCCCCCGGTTAGTACACGTCGTGTTGCACGACAAATAAGGCGAATGATCTAGGTAGAAAAAAAAAAA	170	115
CCCCCCGGCAATAGTATTGAATAGGAGCACACGGCATAAGAGTTTGATACGCCTACAAAAAAAAAAA	168	126
CCCCCCGCTGGTGCATATAGCTTCGTACTACTCAGATAATTGATACAGATGTATATAAAAAAAAAAA	170	114
CCCCCCGGATATATAGTGTTTACTTGTGTTTGATGTCGAAGTGCCGCGCGTAGCCCAAAAAAAAAAA	169	121
CCCCCCTAGAATCTCCTGGACTACTGTCTATGCCTAGGGAGAACGATCAACATTATAAAAAAAAAAA	169	126
CCCCCCATAAAGGAATAAAATGGCTGACCATCCAGCACACGCTCGCTGTCTAGATCAAAAAAAAAAA	163	124
CCCCCCGCATGATCTTGGCGCGGCCGCTATGTCGCCGCCATGCTCACATCCTGGATAAAAAAAAAAA	164	121
CCCCCCGTTAGTAACCGAATGAGTACTGAATATGCTAATCCTGGCAGTTACATCCCAAAAAAAAAAA	168	121
CCCCCCTCTATAGAGACTGGTAACTCGCTGTATAACTCCGCGGCCCGACGATTAATAAAAAAAAAAA	163	127
CCCCCCAGGCTAATGGTCCCCGGGAAGTTGCGGCAGCGAAGTTAGCTCGATACGCCAAAAAAAAAAA	157	123
CCCCCCAGAAACTGGAGAGGACTTCTGTGGCGCTGGTTCAGCGAAGAGGGGCATGCAAAAAAAAAAA	159	126
CCCCCCTAGAAGTAATGGAGGCGAAACAAACAGGTTCGGATAGCCGTGATGTATGTAAAAAAAAAAA	164	119
CCCCCCTCGGAGCCGCGACTCTCGGATAATGTCGACGACCCACGCTATACGTAATGAAAAAAAAAAA	169	119
CCCCCCGGGATACTCGCCGCATTATCGGATGCTTGTAGTCTGACACTTGTGACTCCAAAAAAAAAAA	159	118
CCCCCCAACAGCCGAAGGAAATTACGTCCTTCTCGTGTAAACTCGTCGCCAGCCCCAAAAAAAAAAA	165	124
CCCCCCTTCTAGACCAGGAGTCAGCTACTCGCGAACTGGGACGTACACTATGTACTAAAAAAAAAAA	164	123
CCCCCCATGCCAGTGGTTCCTCGTCAGGGCGTGGGATTGCCGTACATGTACACGCTAAAAAAAAAAA	155	122
CCCCCCGTTGTCCCGCTTGAACAGCTATTGCTCGCGCCACGTCAGCTCCTACGTCGAAAAAAAAAAA	162	121
CCCCCCGGCGGAAATGTTTGTACGCATGCTCAGCGTGCATATCCAGTTTTTCGGACAAAAAAAAAAA	160	126
CCCCCCAAAAGAGCTCTAGTTCTTTATATCAGGTTTCTTCAGCCTCTCCTTCAGATAAAAAAAAAAA	167	125
CCCCCCGAAACGCCTGCGTTCGTCCGCTATCTTGGTATCCACGTTCACCCCTTGATAAAAAAAAAAA	160	124
CCCCCCACATCGCGCTGGGTGCTAGAGAGGTGCATCGGCTTGCCGCGTTGGTTGTTAAAAAAAAAAA	167	113
CCCCCCTTTATCGGACAAGAAGCTGGAACTTCTATATGTACTATGGACGGTAGGGTAAAAAAAAAAA	165	125
CCCCCCGAGTGCATCCGTTGTCAGAACGAGGATCAACCGTCAGGTCAGACCATAGCAAAAAAAAAAA	159	123
CCCCCCTAGTTCCAAGCGCATCCGTTATGGCATTCACATCGCCTGCTTGACTCGTCAAAAAAAAAAA	167	119
CCCCCCTGCTCGAGATGGCGCTCCCAGTCCTACGCCATCCTCAATATGCCATCCTCAAAAAAAAAAA	169	120
CCCCCCGGCAACCTTGCATGGACTCAGAACTGGGGGAGCAGTGGCTATGGAGACCTAAAAAAAAAAA	165	122
CCCCCCGTGGGCCCGCTCCATAGATATAAGGAAATCTCTGCGTAAGTGCACTCCTGAAAAAAAAAAA	153	124
CCCCCCTGACACGCTGATGAGTCGAGTTGGCGAGCCTGGTGAGAGAGGTCGTTGGCAAAAAAAAAAA	169	122
CCCCCCTTGGCGCCTCGCCAGCGGCGCACAGCTCAATGAAGCCAACACTCTCTGACAAAAAAAAAAA	158	123
CCCCCCAACGCACCGCAACAGCAGCATGTCCATGTCTTATGCCTGGTGTGATCCCTAAAAAAAAAAA	169	115

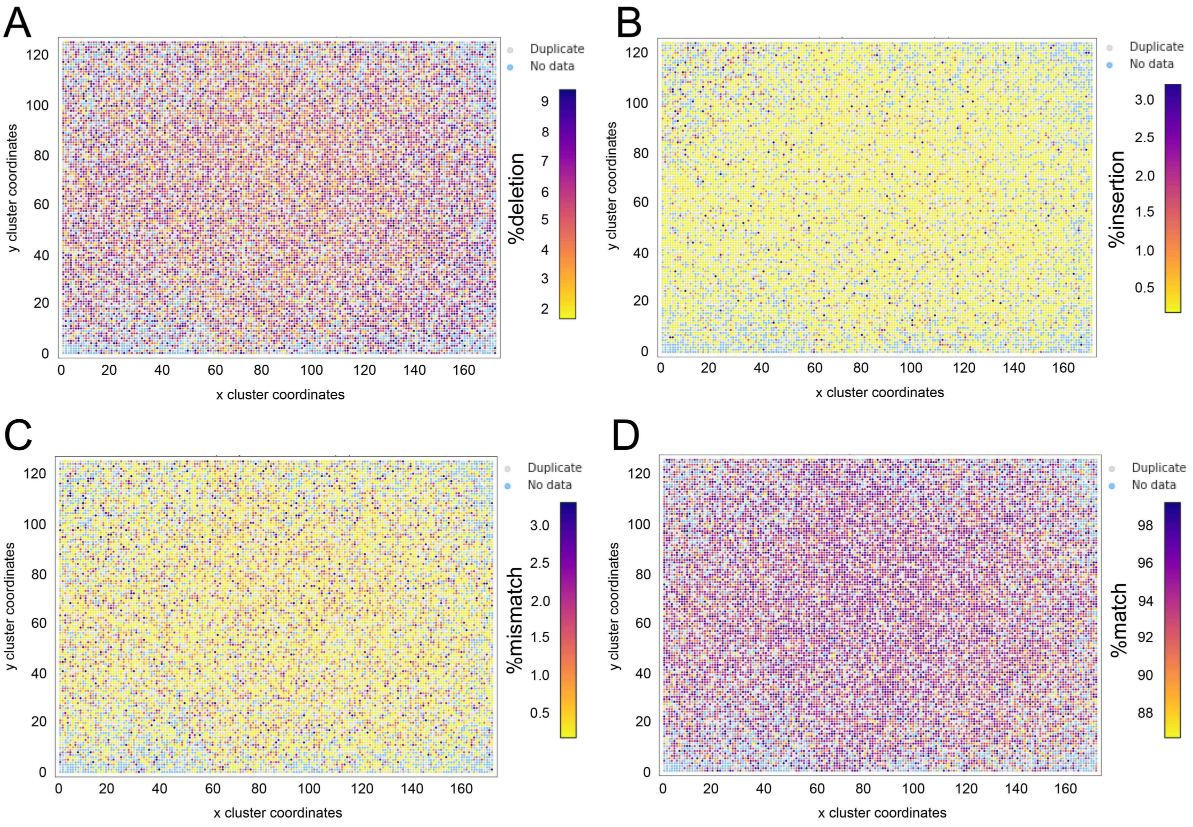

E

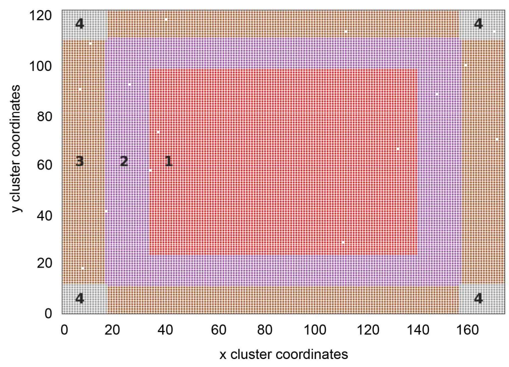

F

	Area 1	Area 2	Area 3	Area 4
% missing cluster	8.93	14.74	23.07	40.11
% match bases	93.31	92.99	92.83	92.56
% deletion bases	4.99	5.2	5.3	5.5
% insertion bases	0.17	0.16	0.19	0.23
% mismatch bases	0.55	0.58	0.56	0.6

Figure S5. Distribution of error-rate as a function of spatial coordinates on the array (4SZ design) A) Deletion rate; B) Insertion rate; C) substitution rate; D) Match rate; E) Synthesis layout subdivided in four areas and the associated error-rates (F).

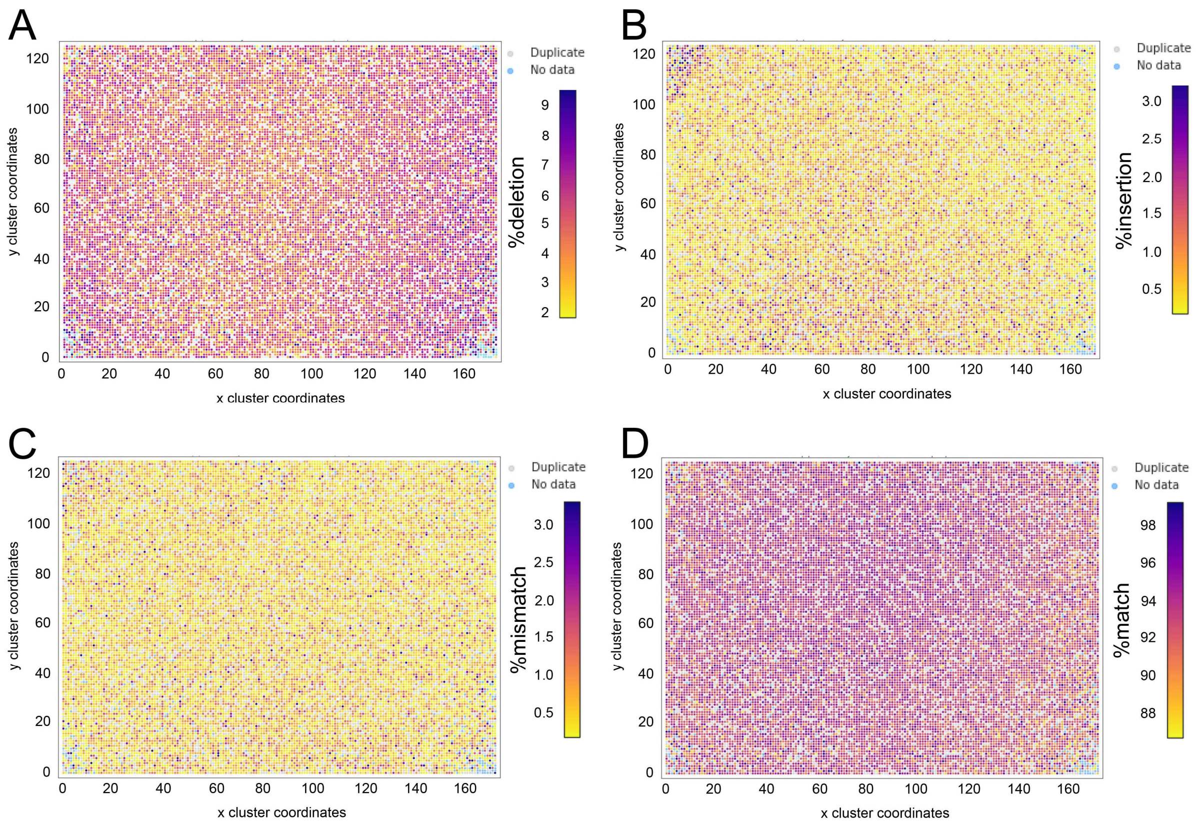

E

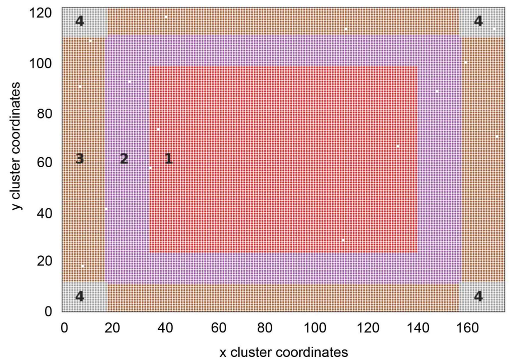

F

	Area 1	Area 2	Area 3	Area 4
% missing cluster	0.5	0.98	1.81	10.59
% match bases	93.19	92.89	92.68	92.37
% deletion bases	4.81	5.01	5.21	5.33
% insertion bases	0.58	0.57	0.53	0.62
% mismatch bases	0.56	0.57	0.56	0.64

Figure S6. Distribution of error-rate as a function of spatial coordinates on the array (capped_2SZ design) A) Deletion rate; B) Insertion rate; C) substitution rate; D) Match rate; E) Synthesis layout subdivided in four areas and the associated error-rates (F).

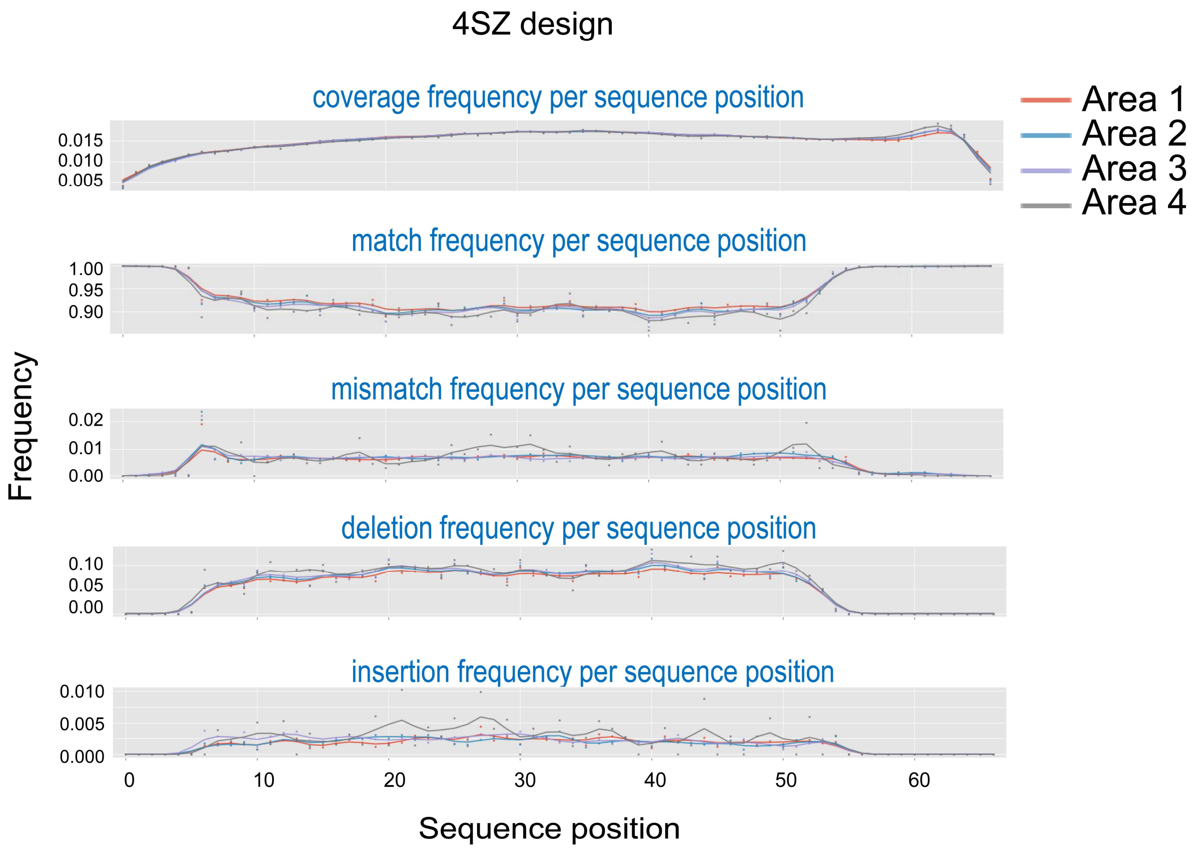

Figure S7. Match and error-rate (% per position) as a function of nucleotide position for all 20000 sequences in the 4SZ design, for all four areas.

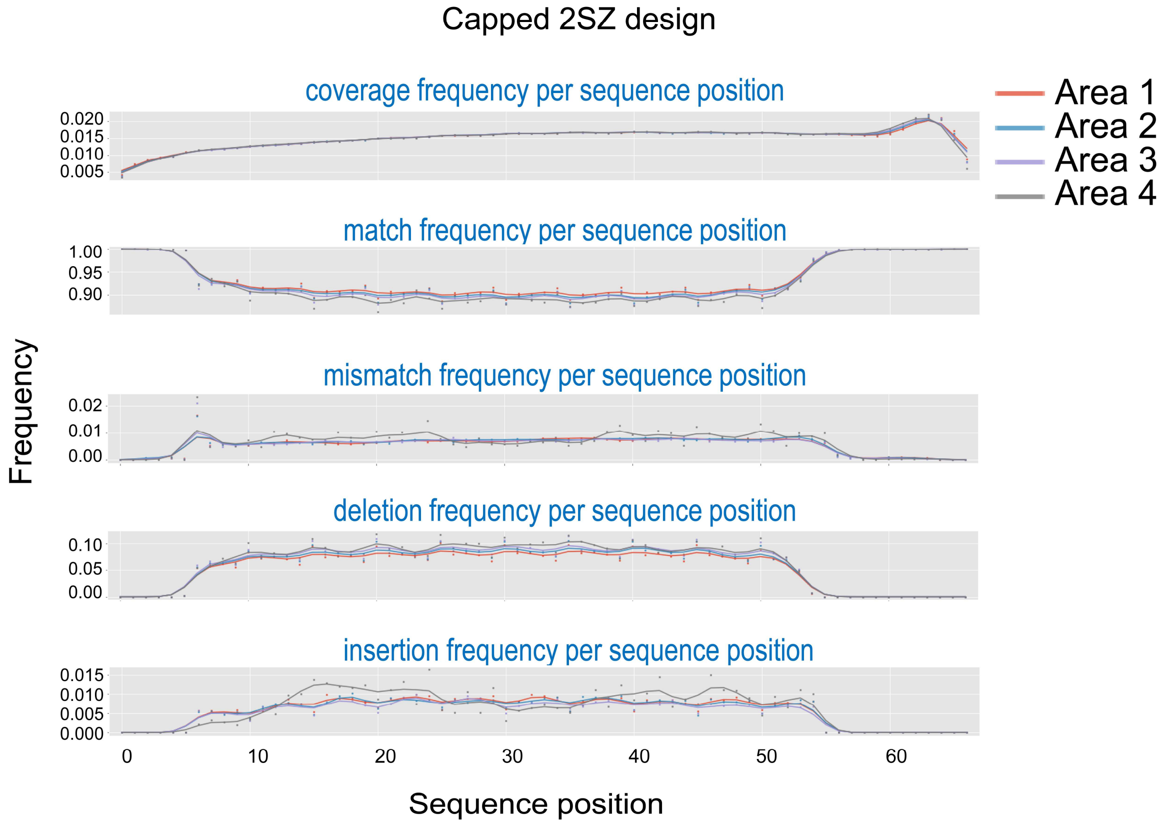

Figure S8. Match and error-rate (% per position) as a function of nucleotide position for all 20000 sequences in the capped 2SZ design, for all four areas.
